# Pattern Triggered Immunity (PTI) in Tobacco: Isolation of Activated Genes Suggests Role of the Phenylpropanoid Pathway in Inhibition of Bacterial Pathogens

**DOI:** 10.1371/journal.pone.0102869

**Published:** 2014-08-07

**Authors:** Ágnes Szatmári, Ágnes Zvara, Ágnes M. Móricz, Eszter Besenyei, Erika Szabó, Péter G. Ott, László G. Puskás, Zoltán Bozsó

**Affiliations:** 1 Department of Pathophysiology, Plant Protection Institute, Centre for Agricultural Research, Hungarian Academy of Sciences, Budapest, Hungary; 2 Laboratory of Functional Genomics, Biological Research Centre, Hungarian Academy of Sciences, Szeged, Hungary; Virginia Tech, United States of America

## Abstract

**Background:**

Pattern Triggered Immunity (PTI) or Basal Resistance (BR) is a potent, symptomless form of plant resistance. Upon inoculation of a plant with non-pathogens or pathogenicity-mutant bacteria, the induced PTI will prevent bacterial proliferation. Developed PTI is also able to protect the plant from disease or HR (Hypersensitive Response) after a challenging infection with pathogenic bacteria. Our aim was to reveal those PTI-related genes of tobacco (*Nicotiana tabacum*) that could possibly play a role in the protection of the plant from disease.

**Methodology/Principal Findings:**

Leaves were infiltrated with *Pseudomonas syringae* pv. *syringae hrcC*- mutant bacteria to induce PTI, and samples were taken 6 and 48 hours later. Subtraction Suppressive Hybridization (SSH) resulted in 156 PTI-activated genes. A cDNA microarray was generated from the SSH clone library. Analysis of hybridization data showed that in the early (6 hpi) phase of PTI, among others, genes of peroxidases, signalling elements, heat shock proteins and secondary metabolites were upregulated, while at the late phase (48 hpi) the group of proteolysis genes was newly activated. Microarray data were verified by real time RT-PCR analysis. Almost all members of the phenyl-propanoid pathway (PPP) possibly leading to lignin biosynthesis were activated. Specific inhibition of cinnamic-acid-4-hydroxylase (C4H), rate limiting enzyme of the PPP, decreased the strength of PTI - as shown by the HR-inhibition and electrolyte leakage tests. Quantification of cinnamate and p-coumarate by thin-layer chromatography (TLC)-densitometry supported specific changes in the levels of these metabolites upon elicitation of PTI.

**Conclusions/Significance:**

We believe to provide first report on PTI-related changes in the levels of these PPP metabolites. Results implicated an actual role of the upregulation of the phenylpropanoid pathway in the inhibition of bacterial pathogenic activity during PTI.

## Introduction

Pattern Triggered Immunity (PTI), also known as Basal Resistance (BR) is a general form of defense that plants use to maintain their integrity by detecting and eradicating almost any type of invading organism, including non-pathogens. PTI is symptomless in most cases; however extensive changes occur at the molecular level. Effector Triggered Immunity (ETI), also known as gene-for-gene resistance of plants, on the other hand requires detection of specific effector proteins of pathogens by plant R-gene products, which eventually leads to the hypersensitive reaction (HR) of plant cells and eradication of that specific pathogen. This manuscript focuses on molecular changes in plant cells during PTI.

Early reports showed that some defence-related genes (PAL – phenylalanine-ammonia-lyase, CHS – chalcone synthase, CHI – chalcone isomerase) are activated after inoculation of *Phaseolus vulgaris* with non-pathogenic *hrp* mutants of *Pseudomonas syringae* pv. *syringae* bacteria as well as with saprobic bacteria [1].

Several authors studied the transcriptomic changes during PTI, mostly in *Arabidopsis*. Navarro et al. [2] treated plants with a fragment of bacterial flagellin (flg22 peptide) and found that the flagellin rapidly elicited (FLARE) genes at 30 and 60 *hpi* are mainly activated, and only a few are repressed. Activation is thought to be achieved by derepression, rather than stimulation. De Torres et al. [3] have found that up to 2 *hpi* transcriptional changes of HR, PTI, and the compatible relationship are mostly identical. Truman et al. [4] therefore sought differences of transcriptional patterns at later time points. At 4 *hpi* the compatible and *hrp* mutant bacteria still caused similar changes. However at 12 *hpi*, the two transcriptional patterns diverged, so they concluded that effectors of the type-three secretion system (TTSS) and other virulence factors of the compatible pathogen have reprogrammed the sensitive plant cell by this time point.

Identification of its elicitors was an important step in the studying of PTI. These so called MAMPs (microbe-associated molecular patterns) are conserved molecules that are indispensable constituents of the bacterial cell. They include flagellin [5], LPS (lipopolysaccharide, [6]), cold shock protein [7] and elongation factor Tu [8]. Identification of the flagellin receptor FLS2 [9] opened the way to determination of downstream signaling events. FLS2 is a leucine-rich repeat receptor-like kinase just like the receptor of elongation factor Tu [10].

Most studies about plant PTI, including the ones mentioned above concentrated on early events of PTI, specialized in deciphering the events of signal perception and transduction, identifying elicitors, their receptors and the downstream signaling events. In contrast there is very little information on the metabolic alterations that eventually protect the plant after PTI was triggered, and hardly anything is known about the mechanisms leading to inhibition of bacterial activity. Therefore in this study we focused on identifying PTI-related genes of tobacco that might possibly play a role in mechanisms leading to inhibition of bacteria.

Plant cell wall fortification is an important factor of mechanical resistance, the formed papillae are thought to constitute a barrier between the plant cell wall and invading microorganisms. Papillae are made up of polyphenolics, callose and a network of cell-wall fortifying glycoprotein matrix mainly consisting of glycin-rich proteins and hydroxyproline-rich extensins [11]. The importance of cell wall fortification and papilla formation is well established in plant-fungal interactions [12]. In the case of bacteria this is speculated to result in a reduced accessibility of nutrients, plus a restricted ability to build up the TTSS and deliver effectors into the plant cell. The possible importance of cell wall fortification and papilla formation in PTI is supported by transcriptional evidence that showed the ability of the compatible *Arabidopsis* pathogen *Pseudomonas syringae* pv. *tomato DC3000* to suppress activation of plant cell wall genes [13], [4]. Hauck et al. [13] found that AvrPto is an effector that suppresses cell-wall based defense in PTI. This was supported by reduced callose staining - an indicator of papilla formation - in Avr Pto transgenic *Arabidopsis* plants.

The phenylpropanoid biosynthetic pathway (PPP) could promote cell wall fortification by lignin synthesis, but it is also an important source of compounds with direct antimicrobial activities. It is not known, whether it plays a role in PTI against bacteria, but its importance in different plant-fungal interactions has long been shown. Tobacco plants with suppressed PAL activity - the second enzyme of PPP - have been shown to be more susceptible to the compatible pathogen *Cercospora nicotianae* [14].

Previous works from our laboratory showed that heat-killed pathogenic bacteria injected into tobacco leaves are able to induce a symptomless local resistance that protects the plant from a following compatible infection, or the hypersensitive response, when re-inoculated with compatible or incompatible pathogens respectively [15], [16]. Later on we found that this local resistance can not only be induced by heat-killed pathogens, but also by saprobic bacteria and some *hrp/hrc* mutants; i.e. pathogens mutated in their hypersensitivity and pathogenicity genes, many of which code for elements of the Type III Protein Secretion System (T3SS) [17]. Based on our previous findings we regard the local resistance of tobacco as a manifestation of PTI. We routinely use the HR-inhibiting effect of PTI as a macroscopical demonstration method. Histochemical [18] as well as transcriptional [19] and proteomic [20], [21] indicators of tobacco PTI have also been established.

*Hrp/hrc* mutants of the *Pseudomonas syringae* pv. *syringae* 61 bacterial strain (original wild type causing brown spot on bean and HR on tobacco) are damaged in their ability to cause disease on bean or HR on tobacco to varying extent, depending on which *hrp* gene is turned off [22]. The *hrcC* mutant is especially useful for investigating PTI, as its ability to induce HR in tobacco is completely abolished, while the induced PTI can nicely be detected by an HR-inhibition test, and it is also a potent elicitor of PTI-related genes [19].

In this study we sought to better understand the molecular mechanisms underlying the physiological and biochemical processes of tobacco PTI. Especially the ones leading to inhibition of bacteria, as the exact way by which the plant achieves symptomless arrest of bacterial growth is yet unknown. At first we identified over 400 EST-s representing genes that are activated during PTI using suppression subtractive hybridization (SSH). Following assembly of the EST sequences into 176 contigs, their transcriptional activation was confirmed by cDNA microarray and real time RT-PCR. A highly represented group in our library, that of the phenylpropanoid genes, was strongly implicated to play an important role in mediating the antimicrobial effects of PTI, as shown by transcriptional (microarray and RT-PCR) data, as well as HR-inhibition assays using pharmacological inhibition of a key enzyme of the phenylpropanoid pathway.

## Materials and Methods

### Plant material

Tobacco plants (*Nicotiana tabacum* cv. *Samsun*) were grown in the greenhouse in soil (general potting mix from peat, cow manure and perlite, pH 6–7). 2 days before and during experiments the 2–2.5 months old tobacco plants were kept in a growth chamber with 16/8 hr light/dark period at 20°C. Hypodermic syringes fitted with a 25 gauge needle were used for the infiltration of the tobacco leaves as described in [23]. At the appropriate time points leaf samples were frozen immediately in liquid nitrogen and stored at −70°C until processing. Non-treated or water-infiltrated leaves were used as control (as indicated in the text).

### Bacterial and chemical treatments

*Pseudomonas* strains were cultured at 28°C on King's medium B [24]. *Pseudomonas syringae* pv. *syringae* 61-1530B strain (kind gift of A. Collmer, Cornell University, Ithaca, USA) was cultured on antibiotic-containing (kanamycin, 50 µg/ml) King's medium B. These bacteria are mutant in their *hrcC* gene, are compromised in their pathogenicity, and are unable to cause HR. Challenge inoculations in the HR-inhibition tests were carried out with *Pseudomonas syringae* pv. *syringae 61* strain, an incompatible wild type strain normally causing HR in tobacco. Overnight cultures of both bacteria were suspended in distilled water and adjusted to 10^8^ CFU/ml before using for infiltration of leaves. PIP (piperonylic acid, Sigma) was dissolved in dimethyl sulfoxide (DMSO) to gain a 200 mM stock solution. Inoculations were carried out with a final concentration of 1 mM. PIP was used either alone, or in combination with *Pseudomonas syringae* pv. *syringae hrcC^−^* (10^8^ CFU/ml) suspension.

Flagellin 22 (flg22) peptide (Genescript, USA) was dissolved in double-distilled H_2_O to a stock solution of 1 mM. This was further diluted to inoculate at a concentration of 1 µM.

### Construction of a subtracted cDNA library enriched for PTI-related sequences

*P. syringae* 61 *hrcC*-treated or non-treated control leaves were ground under liquid nitrogen 3, 6 and 48 hours after inoculation. Equal amounts of 3- and 6-hour samples were pooled. Total RNA was extracted using the Plant Total RNA Extraction Miniprep System (Viogene). mRNA was obtained from 100 µg total RNA for driver and tester each, using the PolyATtract System (Promega) as recommended by the manufacturer.

cDNA production and subtractive hybridization were performed using PCR Select cDNA Subtraction Kit (Clontech) as recommended by the manufacturer. Uninoculated plant material served as “driver” and inoculated plant material as “tester”. Cloning of subtracted fragments was carried out using the TOPO TA Cloning Kit for Sequencing (Invitrogen).

### Nucleotide sequencing and data analysis

425 cDNA clones were individually PCR amplified with universal M13 primers: M13for (-21) TGT AAA ACG ACG GCC AGT; M13rev (-29) CAG GAA ACA GCT ATG ACC. Sequencing of these PCR products was carried out at the sequencing facility of MWG-Biotech Ag. (Ebersberg, Germany). Vector and adaptor sequences were removed and individual sequences were assembled into contigs using SeqMan (Lasergene, DNASTAR). Annotation of contigs was performed using BLAST search [25].

### Construction of a cDNA microarray, hybridizations and data analysis

Construction and use of cDNA microarray was carried out as described [26]. PCR products from the cDNA clones were purified with MultiScreen-PCR plate (Millipore), resuspended in 50% dimethylsulfoxide/water, and arrayed on FMB cDNA slides (Full Moon BioSystems) using a MicroGrid Total Array System (BioRobotics) spotter with 16 pins in a 4×4 grid format. cDNA elements were deposited in duplicates. After printing, DNA was UV crosslinked to the slides with 700 mJ energy (Stratalinker, Stratagene, La Jolla, CA).

For hybridizations, plant total RNA was extracted using Plant Total RNA Extraction Miniprep System (Viogene). Concentration of samples was adjusted to 2 µg/µl RNA using Microcon YM-30 centrifugal filter devices (Millipore). 2.5 µg RNA per sample was reverse transcribed using Genisphere Expression Array 900MPX system (Genisphere) in 20 µl total volume using 20 U of RNasin (Fermentas), 1× first strand buffer and 200 U of RNase H (-) M-MLV reverse transcriptase (Fermentas). All the other probe preparation steps were done according to the manufacturer's instructions (Genisphere). Hybridization was done using Ventana hybridization station (Ventana Discovery), with the buffers provided by the manufacturer. Samples were denatured at 80°C for 5 minutes before applying them onto microarrays. Hybridization proceeded at 55°C for 6 hours in ChipHybe buffer (Ventana). Then 2.5 µl of both Cy5 and Cy3 capture reagent were added to the slides in 200 µl Ribohyb hybridization buffer (Ventana), and incubated at 48°C for 2 h. After hybridization, the slides were washed twice in Ventana Reaction Buffer, then once in 2×SSC, then dipped in 96% ethanol, followed by drying and scanning.

### Scanning of microarrays and data analysis

Hybridized slides were scanned with ScanArray Lite (GSI Luminomics) confocal fluorescent laser scanner at 10 µm resolution. Green laser (543 nm, for Cy3 labelling) and red laser (633 nm, for Cy5 labelling) power were set at 100%, with photomultiplier gain set at 75%. Acquired TIFF files were analyzed using GenePix Pro6.0 software (Axon Instruments Inc.). The “ratio of medians” values calculated by the software were used for calculations. Data corresponding to features with a diameter smaller than 120 pixels and with medians of pixel intensities less than 1.3 times the background intensities in either channels (low signal to noise ratio) were discarded as being non-detectable. Constitutively expressed genes were used for normalization of microarray data, selected and verified by real time RT-PCR. These were ubiquitin-extension protein gene (contig 171, all samples), glycine-rich protein gene (contig 3; 13 copies per microarray, 6 h samples), and SAR8.2c protein gene (contig 11; 8 copies per microarray, 48 h samples).

Statistical calculations were carried out on 3 independent biological replicates. Base 2 logarithmic values of the aforementioned ratios of medians were used for student's T-test. Clones with logarithmic values greater than 1 (p<0.05) were taken as activated.

### MAPMAN analysis

For the MapMan analysis [27] we used the MapMan application for potato created by Rotter et al. [28]. To fit our tobacco data to the available potato mapping file created for the 10 k cDNA microarray of TIGR (The Institute for Genomic Research) we first downloaded the potato gene index (StGI 021005) available from Dana-Farber Cancer Institute (DFCI) and then using the BioEdit Sequence Alignment Editor [29] we selected those tentative potato contigs (TC-s) that are included on the 10 k potato microarray. After that we ran a local BLAST using BioEdit on the database created from these selected potato TC-s to find those TC-s that are most homologous to our PTI-related tobacco contigs. Using the potato TCs and their IDs on the TIGR microarray assigned to the PTI-related tobacco cDNA-s involved on our cDNA microarray we were able to carry out MapMan analysis of our hybridization results.

### Quantitative RT-PCR analysis of gene expression

Total RNA was extracted using the Plant Total RNA Extraction Miniprep System (Viogene) from 0.1 g treated or control leaf material, ground under liquid nitrogen. The concentration of isolated RNA was estimated by measuring its absorbency at 260 nm. 2.5 µg total RNA was reverse transcribed using RevertAid H Minus First Strand cDNA Synthesis Kit (Fermentas) with oligo(dT) primer, according to the manufacturer's instructions. For real-time RT-PCR, gene specific primers were designed using Oligo 6 software (Molecular Biology Insights, Cascade, CO, USA) and synthesized by MWG-Biotech AG (Ebersberg, Germany). Supporting Information S5 presents the sequences of the primers. We used 2.5 µl of a 10-fold dilution of the cDNA stock, in 15 µl reactions. Final concentration of primers was 0.2 µM. PCR was carried out using the iQ SYBR Green 2× Supermix (Biorad), on the DNA Engine Opticon 2 (MJ Research). Cycling parameters were the same for all primers: initial 95°C for 6 min, followed by 40 cycles of 95°C for 30 sec, 60°C for 1 min, plate read step. Measured C(t) values were always normalized against actin (GeneBank X69885) as an internal control. Values of the absolute controls were taken as unit value. Measurements of 3 biological replicates were averaged, standard deviations were calculated. Student's T-test was used to determine, if sample values differ from the water treated control significantly (p<0.05).

### Electrolyte leakage measurement

For electrolyte leakage measurement, middle aged tobacco leaves were pre-treated by injection with 5*10^7^ CFU/ml *P. syringae* pv. *syringae 61 hrcC* mutant bacteria in distilled water; combined with 1 mM PIP (diluted 200× from 200 mM PIP in DMSO stock) or 200× diluted DMSO as a control. Five hours after the pre-treatment leaves were injected with 10^8^ CFU/ml *P. syringae* pv. *syringae 61* (HR-inducing wild type). Sixteen hours after the challenge inoculation (before development of the necrosis), 2 cm diameter leaf discs were cut out. Three leaf discs/sample were floated on 10 ml distilled water and incubated at 100 rpm at 23°C. Conductivity was measured by a conductivity meter (Oakton pH/Con 510, OAKTON Instruments, USA) at different time points after cutting the leaf discs.

### Thin Layer Chromatography (TLC)

Tobacco leaf tissue samples (500 mg) were collected 6 hours after infiltration and were ground with a mortar and pestle under liquid nitrogen. Tissue powder was extracted with 1 ml precooled 90% methanol in microfuge tubes. Samples were then centrifuged at 15000 rpm for 15 minutes. Supernatants were used for quantitative measurements of p-coumaric acid and cinnamic acid by thin layer chromatography (TLC) using 20 cm×10 cm aluminum foil-backed normal particle silica gel 60F_254_ chromatoplates (TLC, #5554 from Merck, Darmstadt, Germany) prewashed by acetonitrile-water 85∶15. TLC separation of the two phenolic acids was carried out with toluene-acetic acid 9∶1 (v/v) as mobile phase in an unsaturated TLC chamber at room temperature. All used solvents were analytical grade purchased from Reanal (Budapest, Hungary).

Test substances p-coumaric acid and cinnamic acid were dissolved in 90% methanol and diluted to give solution containing 1 µg of each in 1 ml. 15 µl of each plant extract and 5–20 µl of solution of test substances (equivalent to 5–20 ng of each) were applied to the adsorbent layer at 10 mm height in 4 mm bands (6 mm space between two bands) with Linomat IV sample applicator (CAMAG, Muttenz, Switzerland).

The developed plates were dried by a cold air stream using a hair-drier (5 min) and the chromatographic spots of p-coumaric and cinnamic acids were evaluated by densitometric measurements by means of a Shimadzu (Kyoto, Japan) CS-930 densitometer at 307 and 274 nm, respectively. Concentrations were calculated as nmol/g (leaf) fresh weight for better comparability of amounts of the two substances.

## Results

### 176 putatively PTI-activated tobacco genes were found by suppression subtractive hybridization

Suppression subtractive hybridization was used to find genes that are activated during PTI. For this purpose one half of the tobacco leaves was treated with a 10^8^ CFU/ml suspension of *P. syringe hrcC* mutant bacteria, while the other half served as a control. We used untreated controls at this stage, as from former experiments it was known that part of the PTI-related genes might be activated to a small extent by wounding; however we did not want to lose these potentially important genes during subtraction. All other downstream experiments (microarray, RT-PCR, etc.) included water treated (i.e. wounded) samples for controls, and further conclusions were only drawn for PTI-related genes verified using water (or DMSO, where appropriate) treated controls.

Samples were taken 6 and 48 hours after inoculation, and at the end of the subtraction process, a cDNA library was obtained that was enriched in cDNA sequences corresponding to activated genes. cDNAs were cloned, resulting in 430 clones from the early sample and 460 clones from the late sample. Half of each clone library was sequenced randomly (425 clones altogether). The sequences with appropriate quality (424) were deposited to dbEST, and can be found in GenBank under accession numbers JZ124214-JZ124637. These sequences were assembled into 176 contigs. 68 of the contigs were made up of 2 or more clone sequences, the rest contained only 1 clone.

**Figure 1:**
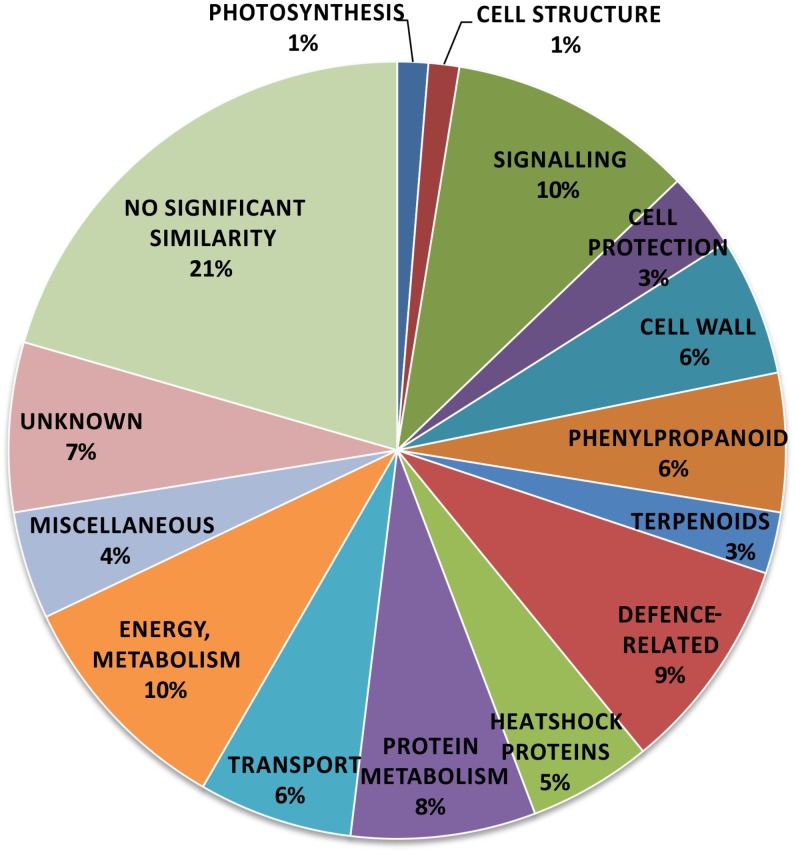
Pie chart representing percent ratios of putative function-based groups of the tobacco genes activated during PTI. 156 putatively PTI-activated tobacco genes were found by suppression subtractive hybridization of samples taken 6 and 48 hours after inoculation of tobacco leaves with *P. syringe hrcC*- mutant bacteria. BLAST nucleotide similarity search (Altschul et al. 1990) was used to assign putative functions to the sequences. Based on the identified putative functions the genes were classified into 15 groups: cell structure (2), signaling (20), cellular protection (7), cell wall (11), phenylpropanoid (9), terpenoid (4), defense related (16), heat shock (9), protein metabolism (13), transport (12), metabolism and energy (21), photosynthesis (2), miscellaneous (7), unknown function (11), no significant similarity (32). The ratios and the corresponding percentages are demonstrated in the figure.

BLAST nucleotide similarity search [25] was used to assign putative functions to the sequences. All sequences were submitted to the DDBJ blastn engine (http://blast.ddbj.nig.ac.jp/blastn?lang=en) as a multiple sequence query. All blast parameters were default (Database = DDBJ ALL nucleotide database; Expect value = 10, Matrix = BLOSUM62). The blast search assigned the 176 contigs to 156 distinct genes, as a few contigs were included in the same genes, without an overlap. A table containing all of the 156 genes and the putative similarities is given in Information S1. The set of identified genes at this stage might have contained some wounding-induced ones, therefore the experiments used to verify PTI-related gene-activation (microarray- and RT-PCR experiments) all contained water treated controls. Based on the identified putative functions the genes were classified into 15 groups: cell structure (2), signaling (20), cellular protection (7), cell wall (11), phenylpropanoid (9), terpenoid (4), defense related (16), heat shock (9), protein metabolism (13), transport (12), energy and metabolism (21), photosynthesis (2), miscellaneous (7), unknown function (11), no significant similarity (32) (Figure 1). The predicted groups of gene functions were determined manually, often searching for the most recent studies on the putative biological function of each gene.

### Activation of 55 PTI-related genes were verified by microarray experiments

To validate the activation of the genes represented by the subtracted clones/contigs, a cDNA microarray was constructed from the sequenced clones. Stringent conditions, as described in Materials and Methods were used for hybridizations to avoid any false-positives although this compromised the sensitivity to a certain extent. Leaves again were infiltrated with 10^8^ CFU/ml suspension of *P. syringe hrcC* mutant bacteria; however controls were water treated here, as well as in all downstream experiments afterwards. Leaves were harvested 6 and 48 *hpi*. Total RNA was extracted, quantified, transcribed and labelled as described in Materials and Methods. Hybridization of PTI-induced 6 *hpi* samples to the cDNA microarrays containing the 176 contigs resulted in 80 detectable contigs (i.e. ratio of medians of pixel intensities significantly divergent from the background, as described in Scanning of Microarrays and Data Analysis in Materials and Methods), of which 52 proved to be activated significantly, i.e. feature signal of the *P. syringe hrcC* mutant-treated sample was at least 2 times that of the water-treated control, tested by student's T-test on data from three independent replicates (p<0.05), (as shown in Table 1). The same with 48 *hpi* samples resulted in 32 detectable contigs, of which 18 were activated significantly. Altogether 55 genes were detected to be upregulated at either 6 *hpi* or 48 *hpi* or both, according to the cDNA microarray.

**Table 1:** cDNA microarray hybridization results of contigs identified from PTI-induced tobacco.

Linear fold changes of PTI-related genes at 6 and 48 *hpi* measured by microarray				
Contig number	6 h fold change	48 h fold change	Genbank accession	Gene name
1	5.51*	3.86*	X74453	*N.tabacum* OMT I-b mRNA
2	2.99	8.02*	AB041519	*N. tabacum* mRNA for P-rich protein, NtEIG-C29
3	0.98	1.56*	Y19032	*N. tabacum* cell wall protein (TLRP tyrosine- and lysine-rich protein)
4	1.34	3.99*	AF151215	*N. glauca* cell-type guard cell glycine-rich protein
5	11.5*	7.51*	AB035125	*Nicotiana tabacum* NtEIG-E17 mRNA for glycine-rich protein, complete cds
6	7.18*	4.98*	AB044153	*Nicotiana tabacum* mRNA for peroxidase, complete
7	4.77*		AB206920.1	*Capsicum annuum* catf2 gene for acyl-transferase
8	5.9*	3.84*	X78203	*H.muticus* mRNA for glutathione S-transferase
10	3.6*		AB041515	*Nicotiana tabacum* NtEIG-E80 mRNA, complete cds
11	2.32*	0.78*	M97360	*Nicotiana tabacum* protein SAR8.2c
12	2.93*		AF082893	*Solanum tuberosum* methionine synthase (MS)
13	1.63*	4.66*	AY422690	*N. attenuata* tissue-type shoot beta-tubulin (TUB)
14	2.23*		Z71395	*N.plumbaginifolia* mRNA for calreticulin
15	2.96		BT013651	*L. esculentum* putative ABC transporter
17	3.49*		BT014186	*Lycopersicon esculentum* clone 133363F
18	2.5*		AF127796	*Capsicum chinense* acyl carrier protein (Acl1)
20	2.8*	3.3*	AB061256	*Solanum tuberosum* mRNA for NADP-dependent malic Enzyme
21	2.03*	4.66*	X70343	*N.sylvestris* mRNA for extensin
22	1.84*	1.95	AJ271872	*N. sylvestris* Ext1.2B gene for extensin
23	1.63*	1.39	BT013251	*L. esculentum* putative ABC transporter
24	2.03*		AB117525	*Nicotiana tabacum* NtMKP1 mRNA for MAP kinase
26	6.47*		AJ309300	*Solanum tuberosum* mRNA for putative membrane protein (poni2 gene).
27	3.06*		BT014512	*Lycopersicon esculentum* clone
28	4.6*		L08830	*Lycopersicon esculentum* BiP (binding protein)/grp78 (glucose-regulated prot.)
29	3.77*		L02124	*Nicotiana tabacum* anionic peroxidase gene
31	7.19*		D86721	*Nicotiana tabacum* DNA for glycine-rich
32	2.93*		AF001270	*Lycopersicon esculentum* cytosolic NADP-malic enzyme (LeME2) mRNA
33	2.11*	0.74*	NA	
34	5.66*		AF307144	*Spinacia oleracea* cytosolic 6-phosphogluconate dehydrogenase (pgdC)
35	5.95*	6.52*	AJ538960	*Nicotiana tabacum* cDNA-AFLP-fragment
37	1.5*		NA	
38	3.39*	2.94*	AY368274	*Nicotiana tabacum* cyclophilin-like (CYP1) mRNA
39	7.19*		AF150881	*Lycopersicon esculentum* x L. peruvianum ferulate-5-hydroxylase (CYP84)
40	6.13*		AJ00321	*Solanum tuberosum* mRNA for hypothetical protein
41	3.52*		AF321140	*Nicotiana tabacum* S-adenosylmethionine synthase
44	2.53*	3.12*	AB041516	*Nicotiana tabacum* mRNA for P-rich protein EIG-I30
46	2.15*		X71441	*N.tabacum* mRNA for cytochrome b5
47	3.32*	6.58*	X60058	*Nicotiana tabacum* blp5 mRNA for luminal binding protein (BiP
48	4.29*	6.98*	NA	
52	8.66*		U57350	*Nicotiana tabacum* epoxide hydrolase mRNA, complete cds
53	0.7*	0.49*	AY904339	*C. annuum* calcium-dependent protein kinase 4 (CDPK4)
54	1.25	2.43*	Z29529	*N. tabacum* ethylene forming enzyme (EFE).
56	1.54		U91723	*N. tabacum* 14-3-3 isoform b T14-3b
57	0.77		DQ016993	*I. batatas* putative L24 ribosomal protein
59	1.48*	3.85*	AF290618	*N. glauca* putative delta TIP (MIP2)
60	1.87*		AK221707	*A. thaliana* MAP3K-like protein kinase
61	2.9*		D26015	*N. tabacum* CND41, chloroplast nucleoid DNA binding protein
62	2.04*	1.9*	X58108	*L. esculentum* mRNA for enolase
63	2.99*		D26460	*Nicotiana glauca* X *Nicotiana langsdorffii* mRNA for tumor-related protein
64	1.73		D17467	*N. tabacumphenylalanine* ammonia-lyase
67	1.33	1.40	X66856	*N. tabacum* MST1 mRNA
68	2.29*	1.73	AY087786	*Arabidopsis thaliana* clone 38412 mRNA, complete sequence
73	4.05*		AF542544	*Nicotiana attenuata* 5-epi-aristolochene synthase
76	1.82*		NA	
77	2.99*		AF082893	*Solanum tuberosum* methionine synthase (MS) mRNA, complete cds
87	1.02		BT012770	*L. esculentum* clone 113748F
102	1.55*		X55974	*N.plumbaginifolia* mRNA for superoxide dismutase
108	1.10		Z29529	*N. tabacum* ethylene forming enzyme (EFE)
111	2.24*		BT004527	*Arabidopsis thaliana* At4g24190/T22A6_20 gene
116	3.14*	3.26*	NA	
122	3.36*		AF001270	*Lycopersicon esculentum* cytosolic NADP-malic enzyme (LeME2)
124	3.07*		U91723	*Nicotiana tabacum* 14-3-3 isoform b T14-3b Mrna
125	2.11*		Y11348	*M. sativa* mRNA for annexin-like protein
126	4.1*		AB007907	*Glycine max.* mRNA for 6-phosphogluconate dehydrogenase
127	1.61		AF370549	*A. thaliana* Putative ribosomal protein (F5A13.4)
128	1.30		NA	
129	2.1*		NA	
130	2.17*		AF004233	*Nicotiana tabacum* hydroxy-methyl-glutaryl-coenzyme A reductase (HMGRL)
132	1.72		AF243180	*L. esculentum* dicyanin
133	2.67*		L14594	*Catharanthus roseus* heat shock protein 90 mRNA,
136	5.91*		X74452	*N.tabacum* OMT I-a mRNA
137	2.8*		AB073628	*Nicotiana tabacum* mRNA for receptor-like protein kinase, complete cds
156	0.72		AJ421413	*N. tabacum* mRNA for alpha-tubulin (tubA3 gene)
161	0.98	0.95	AJ844617	*P. major* mRNA for polyubiquitin (ubq3 gene).
163	2.3*	4.74*	AK121755	*Oryza sativa* (japonica cultivar-group) cDNA
165	1.34	3.71	Z21796	*L. esculentum* chorismate synthase 1 precursor
169	1.18*	0.83	AB012636.	*N. sylvestris* Lhcb1*1 gene for light harvesting chlorophyll a/b-binding protein
170	3.56*	1.30	AF001270.	*Lycopersicon esculentum* cytosolic NADP-malic enzyme (LeME2)
171	1.01	1.15	AJ223329	*N. tabacum* TUQG3 gene, complete CDS.
172	3.25*		U64823	*Nicotiana sylvestris* amino acid permease (nsaap1)

Linear fold changes of the PTI-related genes in tobacco leaves treated with *Pseudonas syringae* pv. *syringae hrcC*- bacteria versus water controls were calculated based on 3 independent biological replicates. Base 2 logarithmic values of the ratios of medians of bacterium- and water-treated controls were used for student's T-test (p>0.05). Significant activation is designated by asterisks.

**Figure 2:**
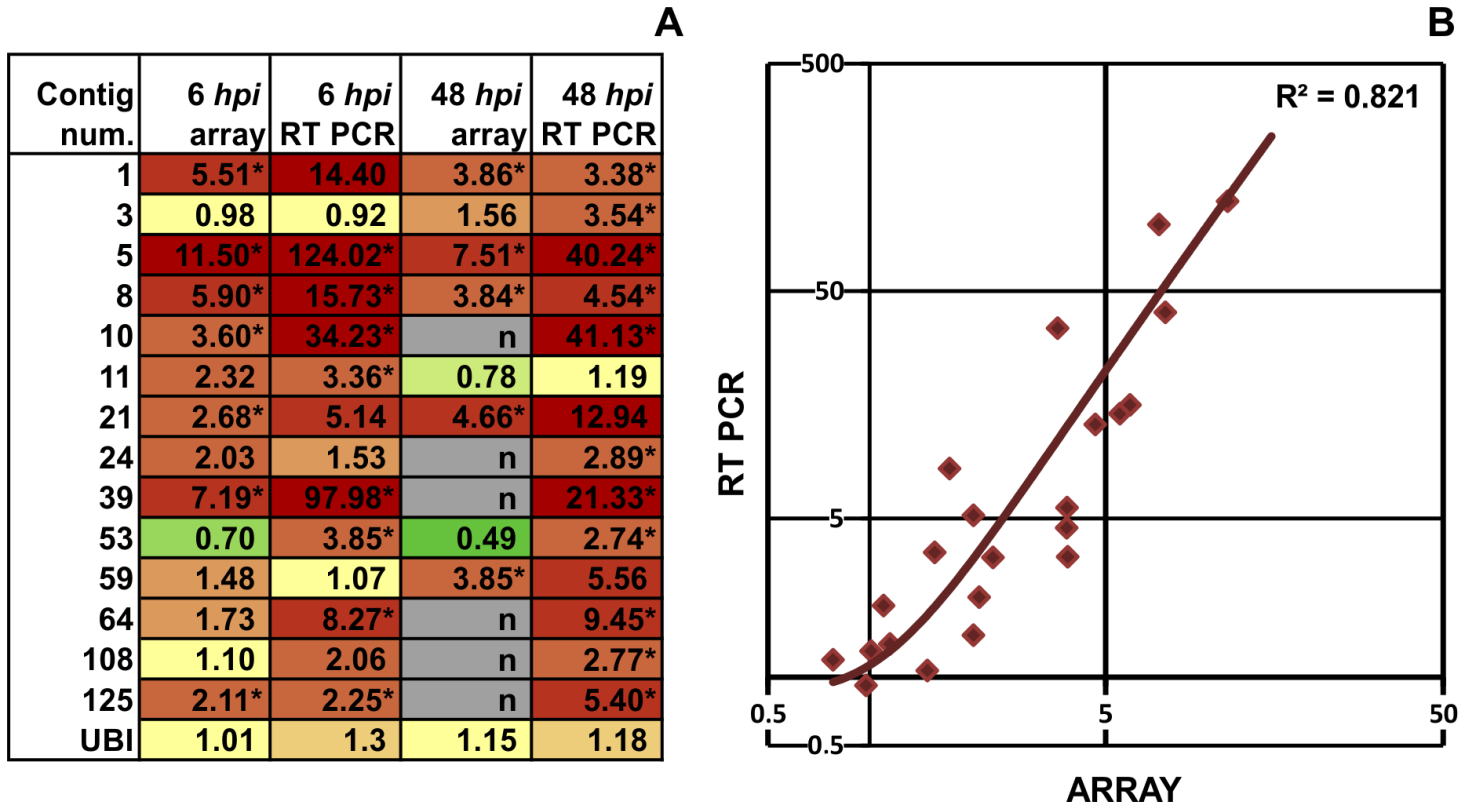
Validation of microarray results by real time RT-PCR. A) Heat map diagram of the expression ratios of randomly selected genes obtained by microarray hybridization and real time RT-PCR. Tobacco leaves were infiltrated with *P. syringae* pv. *syringae hrcC*- bacteria suspension or water, samples were taken 6 and 48 hours later. Values are averages of three independent biological replicates. Stars indicate significant gene activation greater than 2-fold (p<0.05) as compared to water treated controls. Color coding: red: activation, green: repression, yellow: no significant activity change. The letter “n” means no data available. B) Correlation of the RT-PCR and microarray derived expression ratios of the genes from part A). RT-PCR ratios (both 6 and 48 hpi) from the table in Fig. 2A) were drawn on the y axis, while array derived ratios were drawn on the x axis of the graph. Both axes are on a logarithmic scale. The best fitting regression trend line was found to be an order 2 polynomial regression trend line having the highest r-squared (R^2^) value with p<0.01.

A subset of microarray results was validated by real time RT-PCR. Heat-map coloring of the expression ratios in Figure 2A illustrates that in most measurements the direction of the expression change coincided between microarray hybridization and real-time RT-PCR (including 6 *hpi* and 48 *hpi* measurements). There was only one case (contig 53) where hybridization reported repression, while real-time PCR revealed activation. This anomaly might be due to the hybridization of similar/related gene sequences, which was otherwise minimalized by keeping the hybridization conditions adequately stringent. Microarray results correlated well with RT-PCR results (Figure 2B). The best-fitting order 2 polynomial regression trendline had an R^2^ of 0.821. Array-derived ratios tended to be lower than RT-PCR values, which is in accordance with the common experience that cDNA arrays tend to “shrink” the ratio of activation [30].

### MAPMAN analysis reveals substantial differences between early and late phases of PTI

We used MAPMAN (http://mapman.gabipd.org) to visualize the differences between early (6 h) and late (48 h) sets of activated, biotic stress-related genes during PTI. Potato TC-s (Tentative Contigs) could be assigned to 158 PTI-related tobacco contigs based on similarity. Thirty-five of these tobacco contigs were mapped on the biotic stress pathway. The activation data from our microarray hybridizations were then visualized by the potato biotic stress pathway in MAPMAN created by Rotter and coworkers [28] (Information S2).

Members of some groups of genes were particularly activated in 6 h samples, while this activity was attenuated in 48 h samples. These so called early gene groups were the auxin, redox-state, *signaling*, and secondary metabolite related genes, the latter including *phenylpropanoid genes*.

Interestingly, one group, the *proteolysis genes* behaved in the opposite way, showing low activity in 6 h samples, while having strong activity in 48 h samples. Three groups, abiotic stress genes, peroxidase genes and heat shock protein genes remained at a constantly high level of activity.

### Signal transduction-related genes are activated at early and late time points

Signal transduction-related genes were selected for further transcriptional investigation as this group of genes might mean a key to the regulation of the development and action of PTI. The group was highly represented among the identified PTI-related genes, 20 of 176 contigs, *i.e.* 11% of the contigs. Members of the best described signal transduction pathway of plant PTI, the MAPK pathway [31] were found: a receptor-kinase (C90) and two MAP-kinases (C24, C58). Interestingly, contigs of genes representing three calcium-dependent proteins were also found (C53 - calcium-dependent protein kinase 4, C95 - Avr9/Cf-9 rapidly elicited protein 31, C125 - annexin-like protein).

**Figure 3:**
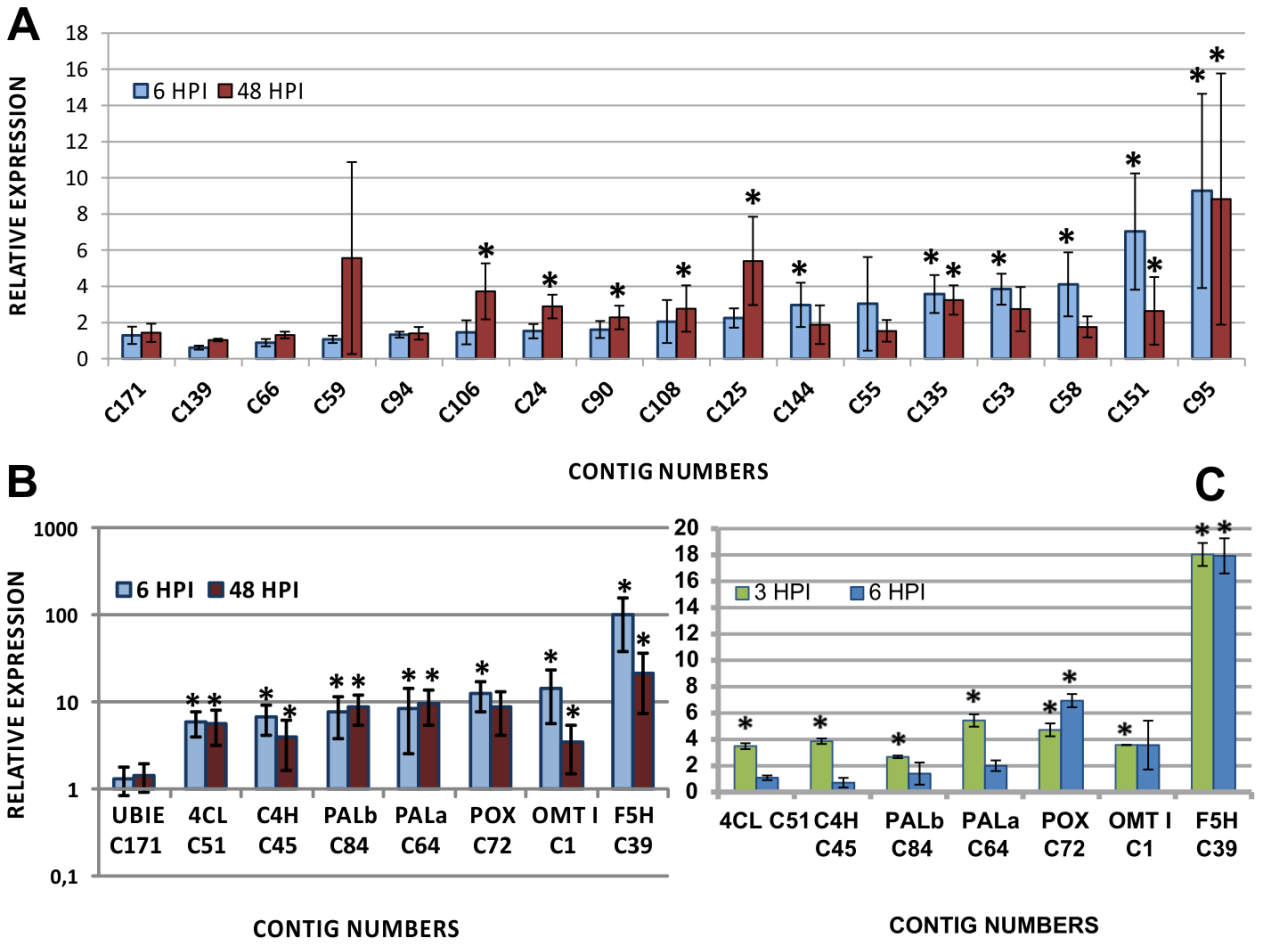
Verification of gene-activation by real-time RT-PCR. A) signal transduction-related genes. B) phenylpropanoid pathway and lignification genes. Ratios of mRNA levels compared to water-treated controls in the order of the strength of activation at 6 hours after treatment. Tobacco leaves were infiltrated with *P. syringae* pv. *syringae hrcC-*suspension, samples were taken 6 and 48 hours later. Values are averages of three independent biological replicates. Each replicate was normalized by corresponding actin levels. Stars indicate significant gene activation (p<0.05) as compared to water treated controls. Contig C171 (ubiquitin extension protein) was used as a constitutive control. C) Flagellin as an elicitor of the phenylpropanoid pathway and lignification genes. Ratios of mRNA levels compared to water-treated controls are indicated. Tobacco leaves were infiltrated with flagellin 22 peptide solution, samples were taken 3 and 6 hours later. Values are averages of three independent biological replicates. Each replicate was normalized by corresponding actin levels. Stars indicate significant gene activation (p<0.05) as compared to water treated controls. Legend A) C139: sphingosine-1-phosphate lyase (SPL); C66: SR1 Nt-rab7b membrane-associated GTP-binding protein; C59: CND41, chloroplast nucleoid DNA binding protein; C94: phosphatidylinositol synthase; C106: putative PTS HPR protein involved in serine-phosphorilation; C24: NtMKP1 MAP kinase; C90: receptor-like protein kinase; C108: ethylene forming enzyme (EFE); C125: annexin-like protein; C144: WD repeat protein AN11; C55: 14-3-3 protein isoform; C135: DNA binding protein Rav; C53: calcium-dependent protein kinase 4 CDPK4; C58: MAP3K-like protein kinase; C151: NtHSF1 heat shock transcription factor; C95: Avr9/Cf-9 rapidly elicited protein 31 (ACRE31). Legends B) and C) PAL, Phenylalanine ammonia-lyase; C4H, cinnamate 4-hydroxylase; 4CL, 4-hydroxycinnamoyl-CoA ligase; OMT I, O-methyltransferase; POX, peroxidase; F5H, ferulate 5-hydroxylase.

Real time RT-PCR investigation of each member of the above two groups (MAPK pathway, calcium-dependent proteins) proved significant elevation in expression either at 6 *hpi* or 48 *hpi* or at both, as compared to controls injected with water (Figure 3A and Information S3). Altogether, 11 out of 16 contigs representing various signaling-related genes were significantly activated according to real time RT-PCR measurements.

### Complete network of phenylpropanoid genes is activated in PTI-induced leaves

**Figure 4:**
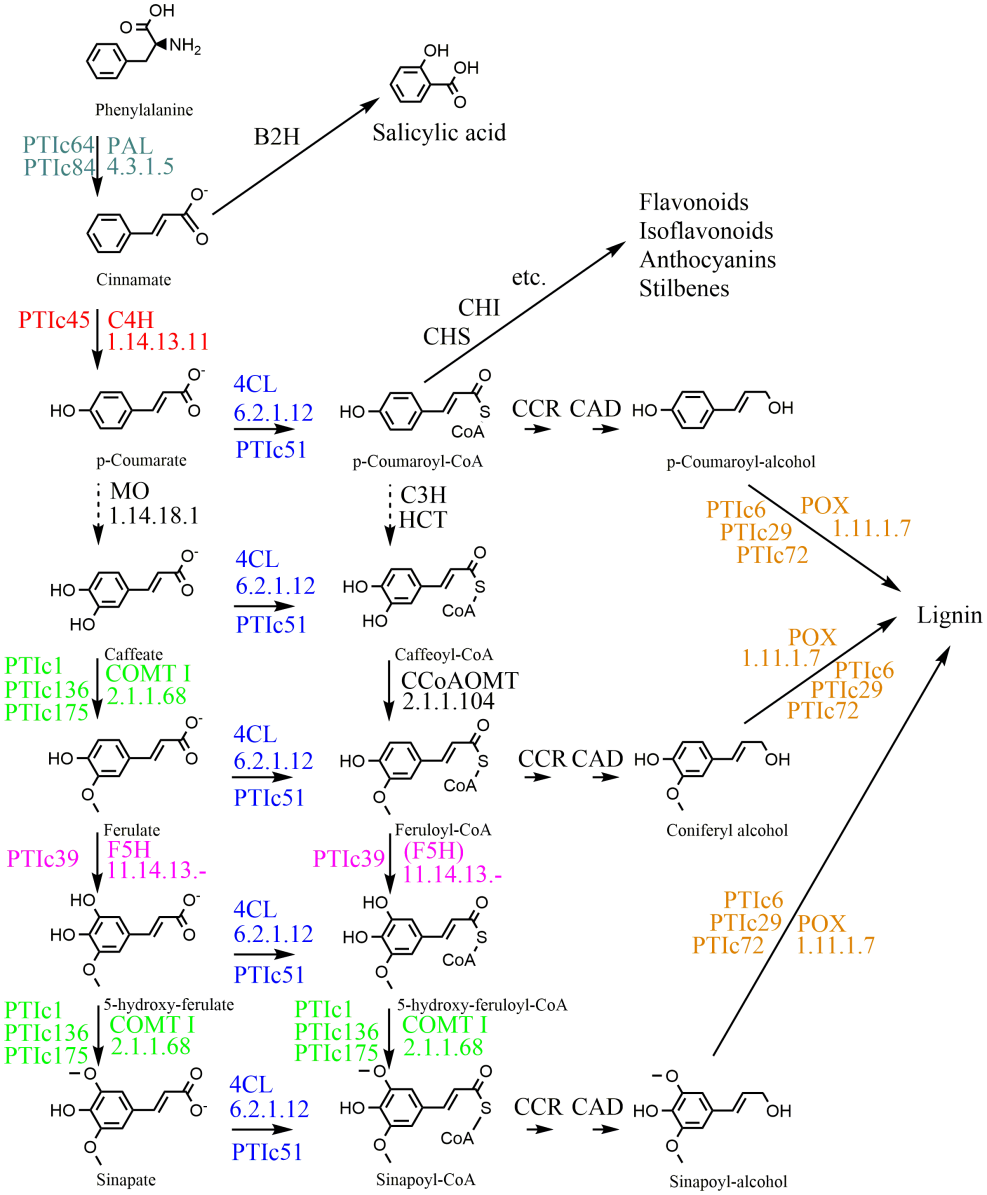
A current view of the phenylpropanoid metabolism. Colours indicate the enzymes found in our PTI cDNA library. Each colour corresponds to a specific type of enzyme. Numbers of contigs, and KEGG codes of each enzyme are indicated (Kyoto Encyclopedia of Genes and Genomes http://www.genome.ad.jp/kegg/). Dashed arrows indicate that the process mechanism of the given enzymatic reaction is not known. Abbreviations: PAL, Phenilalanine ammonia-lyase; C4H, cinnamate 4-hydroxylase; MO, monophenol oxidase; COMT I, caffeic/5-hydroxyferulic acid O-methyltransferase; F5H, ferulate 5-hydroxylase; 4CL, 4-hydroxycinnamoyl-CoA ligase; C3H, p-coumarate 3-hydroxylase; HCT, hydroxycinnamoyl-transferase; CCoAOMT, caffeoyl-CoA O-methyltransferase; CCR, cinnamoyl-CoA reductase; CAD, cinnamyl-alcohol dehydrogenase; POX, peroxidase; CHS, chalcone-synthase, CHI, chalchone-isomerase; B2H, benzoic acid-2-hydroxylase. (Based on [54])

Interestingly, a complete network of the phenylpropanoid pathway (PPP) genes leading towards synthesis of lignin-type polyphenols was found in our PTI-related subtracted library. Each PTI-activated PPP gene is indicated with coloured letters in Figure 4, showing that nearly all of the enzymatic steps leading to lignification are represented. Real-time RT-PCR measurements confirmed that all of these genes are significantly activated during PTI (Figure 3B and Information S3). All of the measured gene activities were significantly elevated at 6 *hpi*, and remained above baseline to different extent at 48 *hpi* as well. Activated genes included early genes of the phenylpropanoid pathway such as two phenylalanine ammonia-lyase (PAL) genes, a cinnamic acid 4-hydroxilase (C4H), and a 4-coumarate:CoA-ligase (4CL). Activation of the genes encoding the enzymes of the next stages of the pathway, such as ortho-methyltransferases (OMT) and ferulate-5-hydroxylase (F5H) was also significant. Three isoenzymes of the peroxidases that oxidize the aromatic alcoholic compounds into lignins were also activated.

Real Time RT-PCR also confirmed activation of the same genes by the most examined elicitor of PTI, the flg22 peptide (1 µM) (normalized with actin and a water treated control), as indicated in Figure 3C and Information S3. Magnitude of this activation was lower and seemed to fade away earlier than in *P. s. hrcC*- treated samples. Therefore in this case samples were taken 3 and 6 hours after inoculation (instead of 6 and 48 hours).

### Inhibition of C4H activity decreased the effectiveness of PTI

To assess if disturbing the phenylpropanoid pathway causes a noticeable change in the efficiency of PTI we chose to inhibit C4H. This enzyme, second just after PAL, catalyzes a rate limiting reaction, causing a *bottleneck effect*, so its inhibition will have an impact on all further reactions. Piperonilic acid (PIP) was chosen, known to be a selective, quasi-irreversible inhibitor of C4H [32]. Although PIP is toxic to bacteria to a certain extent, it is metabolized fast *in planta* [32]. Our *in vitro* co-incubation experiments showed that proliferation of *P. syringae* pv. *syringae 61* incubated in 1 mM PIP solution for 30 minutes was lowered by two orders of magnitude. In a dilution series of PIP, 50 µM was the first concentration not to influence bacterial proliferation (data not shown). We tested if these characteristics could affect our planned experiments and found that it had no adverse effects on HR-development if bacteria were injected at least 2 hours after PIP administration (Information S4 A). Only when the incompatible *P. syringae* pv. *syringae 61* was co-infiltrated with PIP (i.e. 0 hpi) did we detect a lack of HR, possibly because of inhibition of bacterial activity by PIP. Therefore after considering several possible C4H inhibitors from literature, PIP seemed to be the most suitable for our experiments because of its selective and quasi-irreversible nature.

**Figure 5:**
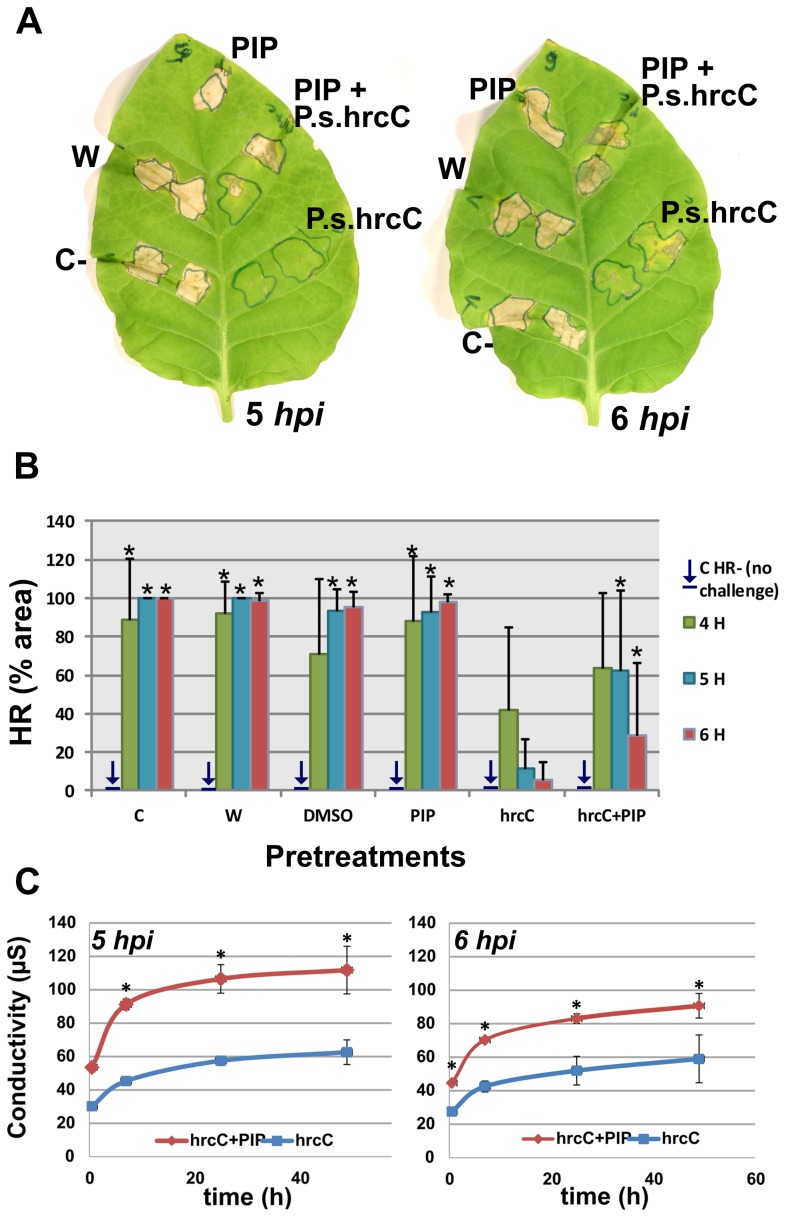
HR-inhibition tests and electrolyte leakage measurements to show the ability of PIP to reduce PTI efficiency. A) Representative images showing the difference in the extension of the HR lesion induced by *P. syringae 61* challenge inoculation following pretreatment with *P. syringae hrcC*- bacteria (hrcC), verus pretreatment with PIP+hrcC, and controls. B) Numerical evaluation of the data. Whole intervein areas were pretreated with piperonylic acid (PIP), *P. syringae hrcC*- bacteria (hrcC), their combination (PIP+hrcC), or as controls: with dimethyl sulfoxide (DMSO), water (W) or left untreated (C). Challenge treatments with *P. syringae 61* (HR-inducing wild type) bacteria followed after 4, 5 and 6 hours. The area of the challenge infiltration was circumscribed with a marker pen. The percentage of this area where HR developed, was noted. Values are the average of three independent experiments. Standard deviations are included in the diagram, and stars indicate significant difference (p<0.05) as compared to the hrcC-treatment. C) Electrolyte leakage measurements to quantify degree of HR development. *P. syringae hrcC*- bacteria (hrcC) alone or in combination with piperonylic acid (PIP+hrcC) were injected into tobacco leaves. 5 and 6 hours after the pre-treatment, leaves were injected with *P. syringae* pv. *syringae 61* (HR-inducing wild type). Leaf disks were cut out 16 hours later, floated on double distilled H_2_O, and conductivity was measured in a time series. Significant differences between hrcC and PIP+hrcC conductivity values are indicated by stars (p<0.05).

The HR-inhibiting feature of PTI was used as an indicator of PTI efficiency. A pretreatment of tobacco leaves with *P. syringae* pv. *syringae hrcC*- prevented development of HR upon a second infiltration (4, 5 or 6 hours later) with the incompatible strain *P. syringae* pv. *syringae 61*, as expected [17]. When PIP was injected into tobacco leaves in combination with *P. syringae* pv. *syringae hrcC*- as pretreatment, the challenge infiltration with the incompatible strain *P. syringae* pv. *syringae* 61 resulted in HR, but a significantly less intensive one, than in the case of the HR-positive controls. This result indicated that the PTI induced by *P. syringae* pv. *syringae hrcC*- bacteria was less efficient when PIP was used to inhibit a key phenylpropanoid enzyme, the C4H. The longer the time elapsed between the pre- and secondary treatment, the more explicit was this difference, being most pronounced at 5 hours in our experimental settings (Figure 5). At the 6-hour challenge treatment the difference in the extent of HR was smaller again between *P. s. hrcC* and *P. s. hrcC*+PIP pretreated interveinals, because PTI was already relatively stronger in the hrcC+PIP treated areas.

To verify the results obtained by visual evaluation of the HR-inhibition tests we carried out electrolyte leakage measurements of the two most important pretreatments: *P. s. hrcC* and *P. s. hrcC*+PIP. Challenge infiltration with incompatible *P. syringae* pv. *syringae* 61 followed 5 and 6 hours later. Shortly before expected HR and tissue necrosis (16 hours after challenge inoculation) leaf disks were cut out and floated on water. Conductivity was measured as a function of time, as indicated in Figure 5C. Results were consistent with the HR-inhibition tests. Conductivity (corresponding to electrolyte leakage and HR) was higher both in the 5-hour and the 6-hour *P. s. hrcC*+PIP samples. The difference between the two pretreatments was greater in the 5-hour samples, just as seen in Figure 5B.

To rule out the possibility that a direct inhibitory action of PIP on the *P. syringae* pv. *syringae hrcC* bacteria used for pretreatment caused the detected difference in the extent of PTI development, we repeated the HR-inhibition experiment with heat-killed *P. s. hrcC* and heat-killed *P. s. hrcC*+PIP as pretreatments. Results were similar with heat-killed as with live *P. s. hrcC* pretreatments, i.e. addition of PIP caused diminished HR upon challenge inoculation with incompatible strain *P. syringae* pv. *syringae* 61 (representative leaf image: Information S4 B).

### Production of p-coumarate is strongly induced by PTI elicitors, and is inhibited by PIP

**Figure 6:**
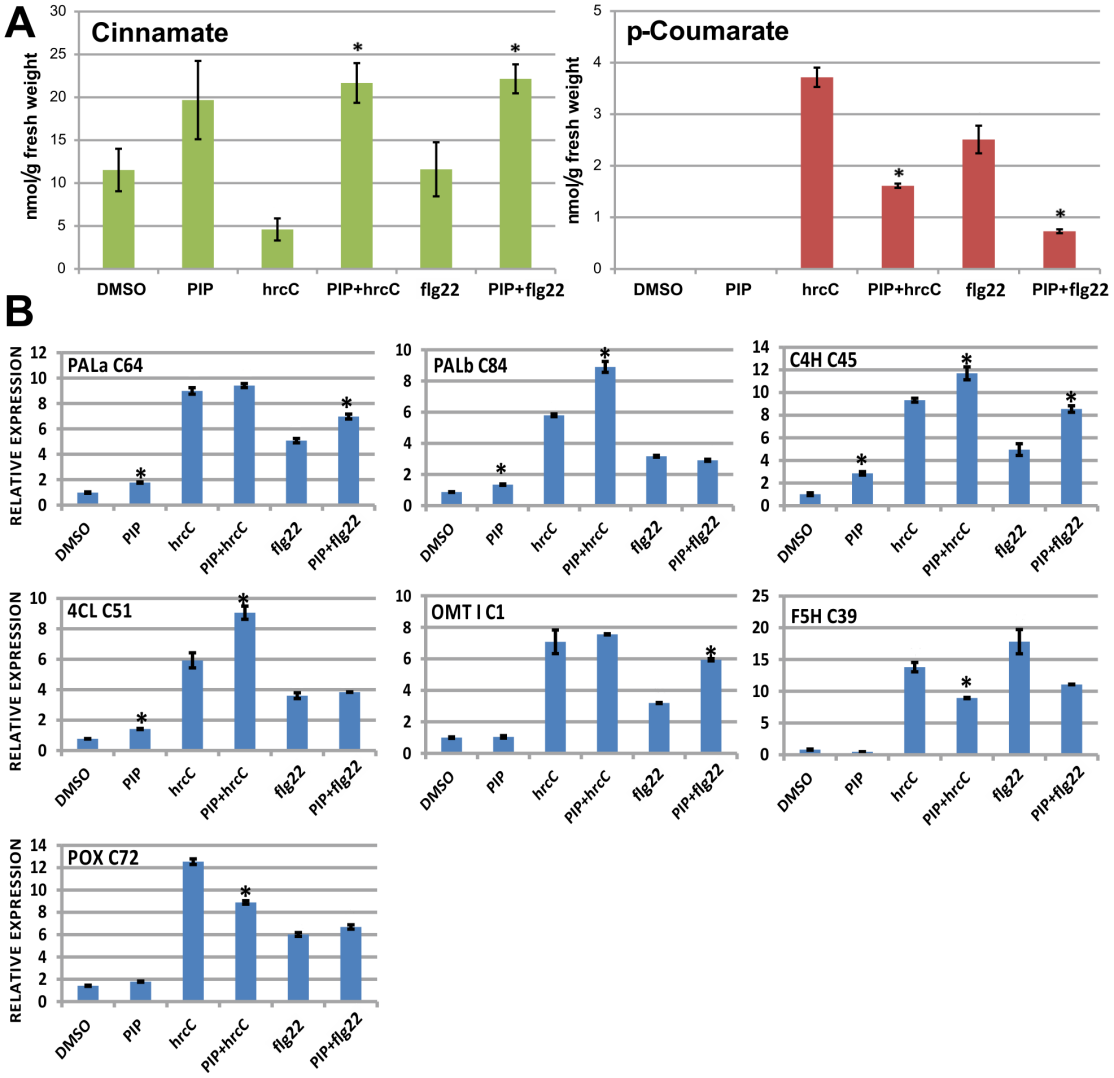
Changes in cinnamic acid and p-coumaric acid metabolite levels, and transcript levels of the related PPP enzymes in PTI-induced, PIP inhibitor-treated, and control tobacco leaf tissue. A) Thin Layer Chromatography (TLC) separation and densitometric measurement of two phenolic compounds, cinnamic acid and p-coumaric acid in PTI-induced and control tobacco leaf tissue. Tobacco leaves were treated with dimethyl sulfoxide (DMSO), piperonylic acid (PIP), *P. syringae hrcC*- bacteria (hrcC), their combination (PIP+hrcC), flagellin 22 peptide (flg22) and combination of piperonylic acid and flagellin 22 peptide (PIP+flg22). Tissue samples (500 mg) were collected 6 hours after infiltration. Separation and detection of p-coumaric acid and cinnamic acid was carried out by thin layer chromatography (TLC) and UV densitometry. A standard line was created using commercially available p-coumaric acid and cinnamic acid to quantitate the two substances in the measured samples. Standard deviations are included in the diagram, and stars indicate significant difference between PIP+PTI elicitors (PTI+hrcC or PTI+flg22) versus elicitor only treatments (hrcC or flg22 respectively) (p<0.05). B) Measurement of gene-activation of phenylpropanoid pathway and lignification genes by real-time RT-PCR. Tobacco leaves were treated with dimethyl sulfoxide (DMSO), piperonylic acid (PIP), *P. syringae hrcC*- bacteria (hrcC), their combination (PIP+hrcC), flagellin 22 peptide (flg22) and combination of piperonylic acid and flagellin 22 peptide (PIP+flg22). Tissue samples (500 mg) were collected 3 hours after infiltration. Ratios of mRNA levels compared to water-treated controls are indicated. Values are averages of three replicates. Each replicate was normalized by corresponding actin levels. Stars indicate significant difference between DMSO control and PIP; furthermore between elicitor only treatments (hrcC or flg22) versus PIP+PTI elicitors (PTI+hrcC or PTI+flg22 respectively) (p<0.05). PAL, Phenylalanine ammonia-lyase; C4H, cinnamate 4-hydroxylase; 4CL, 4-hydroxycinnamoyl-CoA ligase; OMT I, O-methyltransferase; POX, peroxidase; F5H, ferulate 5-hydroxylase.

Thin layer chromatography (TLC) was applied to measure the levels of cinnamic acid, the second phenolic acid in the phenylpropanoid pathway and precursor of p-coumaric acid, moreover substrate of the key rate limiting enzyme of the pathway: C4H (Figure 6A). Levels of the product, p-coumarate were also measured in tobacco leaf tissue in response to treatments with PTI inducing bacteria (*P. syringae* pv. *syringae hrcC*, 10^8^ CFU/ml) and the elicitor flagellin peptide (Flg22, 1 µM), and the C4H inhibitor PIP (1 mM).

Cinnamic acid was present at a basic level of cca. 11 nmol/g fresh weight in our samples, as seen in DMSO controls (Figure 6A). This level was generally elevated upon any type of treatment with the C4H inhibitor PIP, either alone or combined with the PTI elicitors. Elevation compared to control (DMSO) treatment was not significant in the case of PIP treatment alone, but it was definitely significant when PIP was combined with either PTI elicitor. Treatment with PTI inducing bacteria (*P. syringae* pv. *syringae hrcC*-) alone caused significant decrease in cinnamate levels (about halved the concentration) as expected, while treatment with flg22 peptide maintained cinnamate quantity at the basic level of 11 nmol/g fresh weight in the measured 6 *hpi* samples. Looking at pairs of treatment X and treatment X+PIP, the largest difference was found between hrcC and hrcC+PIP treatment among our experimental settings, and difference between flg22 and flg22+PIP was also significant. Knowing that PAL, the previous enzyme in the phenylpropanoid pathway is also activated upon PTI elicitor treatment, one might hypothesize that when elicitors are added, PAL creates elevated levels of cinnamate, which is then normally turned into coumarate by C4H. This transformation is then stuck by the inhibitor PIP, resulting in high levels of cinnamate.

p-Coumaric acid is directly synthetized from cinnamic acid in plants by C4H. p-Coumaric acid (Figure 6A) was not detectable in samples inoculated only with DMSO or PIP (negative controls), as opposed to the other samples inoculated with either one of the PTI elicitors *P. syringae* pv. *syringae hrcC*- (3.7 nmol/g fresh weight) or flg22 peptide (2.5 nmol/g fresh weight). However, when PIP was added to the elicitors, concentration of p-coumarate was measured to be less than half at 6 *hpi*.

TLC experiments with heat-killed *P. s.* hrcC and heat-killed *P. s.* hrcC+PIP gave similar results to those with live *P. s.* hrcC or with the flg22 peptide. That is, coumaric acid was formed in heat-killed *P. s.* hrcC treated samples, and much less of it was detected when PIP was also added (i.e. in heat-killed *P. s.* hrcC+PIP treated samples).

Transcript levels of phenylpropanoid enzymes were also measured in this experiment (Figure 6B). We were curious about any (positive or negative) effect of PIP addition on transcriptional levels of PPP genes. We would have liked to see how changes in transcriptional levels relate to changes in metabolite levels in Figure 6A. We expected that if PIP is supposed to inhibit PTI through inhibiting C4H, than the plant might try to counteract its effect on a transcriptional level. This would mean that PIP addition would elevate transcriptional levels of some PTI-related PPP genes, or at least of C4H.

Samples were taken 3 hours after infiltrations with *P. syringae* pv. *syringae hrcC*- (10^8^ CFU/ml), flg22 (1 µM) and PIP (1 mM), as the investigated genes were shown to be significantly activated by flg22 at this time point (Figure 3). Values in Figure 6B were normalized against actin and water treated control, like in the other RT-PCR measurements. In general, we saw again that *P. s. hrcC*- caused stronger activation than flg22. In the case of 5 out of 7 phenylpropanoid genes (PALa, PALb, C4H, 4CL, OMT-I), addition of PIP to the PTI elicitors caused a slight elevation in gene activity. Interestingly, these 5 genes are the closer ones to phenylalanine, i.e. to the origin of the phenylpropanoid pathway, as can be seen in Figure 4. Even PIP on its own caused a slight elevation in activities of the first 4 PPP genes (PALa, PALb, C4H, 4CL), of which transcription of the C4H gene was most elevated (cca. 3 times).

## Discussion

### Suppression subtractive hybridization to isolate genes having a role in PTI

Tobacco is a well-established model plant for plant-microbe interaction studies, especially in the field of plant-bacterial interactions and a good model plant of the *Solanaceae* family. Plant science results should not be generalized incautiously, as differences between plant-groups always occur. For example in the study of Jakobek and co-workers [1] [33], bean was used as a model plant and chalcone synthase and chalcone isomerase as indicator genes of PTI, besides a PAL and a chitinase gene. However the first two indicator genes seem to be specific for leguminous PTI, as they are activated in *Medicago* as well [34] but not in *Arabidopsis* [4] and tobacco (Szatmari et al. unpublished).

Several authors ([2] [3] [4] [35]) studied the transcriptomic changes during PTI, mostly in *Arabidopsis* model plants. Although exact parameters (bacterium strains, times of sampling) are different, the numbers of activated genes are comparable. Treatments inducing PTI *in planta*, such as injection of flg22 peptide or infection with *P. syringae* pv. *tomato* DC3000 *hrpA* resulted in activation of 220–250 genes within 0.5–7 hours according to the above authors. Our study identified 176 PTI-related tobacco genes assembled as contigs from 425 sequenced clones. So the suppression subtractive hybridization method in our hands resulted in a set of useful data comparable to that generated by microarray experiments. We have to mention that our dataset might include some wounding-related genes, as non-treated control leaves were used for subtraction initially. The interplay between PAMP- and DAMP- (Damage Associated Molecular Pattern) induced signaling and defence mechanisms has been characterized, and some common and also some distinct features have been found [36]. For example flg22 and OG (oligogalacturonides, typical example of DAMP) induced overlapping early transcriptional changes in *Arabidopsis*, but the number of activated genes and the amplitude of these changes was significantly higher after flg22 treatment than after OG treatment. Late responses were on the other hand largely different between the two elicitor types [37].

However, this time we were interested solely in PTI, so we were very strict on verifying PTI-relatedness of as many genes as possible, using only water- (or DMSO-) treated controls in all of the downstream experiments, including microarray and RT-PCR studies.

It is worth mentioning the group identified as “no significant similarity” genes. 32 out of 176 contigs - that is 18% - fell into this category. These could represent important genes that have not been investigated previously. Reasons for this could include that they are low-copy number genes, or genes expressed only in special situations (eg. specific bacterial treatments). Even genome projects cannot guarantee identification of all expressed genes of an organism, as algorithms used to filter genes might miss some gene groups, as happened with more than 300 defensin-like genes in *Arabidopsis*, being shorter than the shortest ORF length programmed in the annotation algorithm at The Arabidopsis Information Resource [38].

### MAPK and calcium signaling related genes in the PTI library

Unraveling the signaling pathways involved in PTI would bring us closer to the possibility of breeding or engineering plants with a higher capacity of PTI. Some authors have already made important steps towards this task. Asai et al. [31] followed a complete MAPK cascade in *Arabidopsis* from the perception of the MAMP flagellin by its receptor-like-kinase-type receptor FLS2 to the corresponding transcription factors. However, genetic evidence later showed that the first element, MEKK1 in fact belongs to another module: MEKK1-MKK1-MPK4 [39], [40]. The MEKK element linking FLS2 and MKK4/5 remains yet to be identified [41], [42]. We have found some elements of a putative tobacco PTI MAPK cascade. Our data however do not allow us to establish the existence of actual interactions between these elements; so they remain as a starting point to find their interactors in future experiments.

Another group of our activated signaling genes during PTI were related to Ca^2+^ mediated signaling. Only a few examples of MAMP-induced elevation of intracellular Ca^2+^ levels exist in plants so far [43]. De Torres et al. [3] showed that a sustained elevation of plant intracellular Ca^2+^ levels occurs in an apparently *avr*-dependent manner. Earlier work from the same laboratory [44] however showed the existence of an early peak of intracellular Ca^2+^ level, which was triggered by bacteria mutant in the type III secretion system (PTI-inducing) as well as incompatible strains. This common early peak could possibly be related to onset of PTI. Ali et al. [45] found that the MAMP LPS, just like avr gene products is capable of generating Ca^2+^-influx into plant cells. Moreover Aslam et al. [46] showed that the MAMPs flg22 and elf18 cause an increase in plant intracellular Ca^2+^ levels 1–4 minutes after treatment.

### PTI development and the phenylpropanoid pathway

Genes related to formation of cell wall deposits were highly represented in our PTI-related cDNA library. These were genes of the phenylpropanoid pathway and also some genes of glycine-rich and proline-rich proteins. Our PTI-activated phenylpropanoid genes covered a major part of the phenylpropanoid pathway (Figure 4). Real time RT-PCR confirmed both early (6 *hpi*) and late (48 *hpi*) activation of these genes (Figure 3B). To assess if activation of these genes can definitely be linked to PTI, we tested gene activation in response to the flg22 peptide as well. Among our experimental settings, each gene was activated (Figure 3C), however to a lesser extent than in response to the *P. syringae* pv. *syringae hrcC*- bacteria (Figure 3B). The response to flg22 seemed to fade away earlier as well. At 3 *hpi* each investigated phenylpropanoid gene was activated significantly, while at 6 *hpi* only two of them: POX (C72) and F5H (C39). One probable reason for this difference might be that whole bacterial cells contain many other PTI elicitors resulting in a stronger PTI reaction than flagellin alone. Higher concentrations of flagellin might also trigger stronger responses, which we did not investigate at present. Still another explanation might be that response to flg22 occurs much quicker, so that peaks of gene activation might occur earlier.

In a northern blot of Zhang et al. [47] *P. tabaci* hrcC- triggered a strong response to three representative genes in *Arabidopsis* at 6, 12 and 24 *hpi*, while the same genes were strongly activated at 3 *hpi* after flg22 treatment, but activation has declined already at 6 *hpi*. Zhang et al. worked with hrcC concentrations of 10^6^ CFU/ml and flg22 concentrations of 1 µM. For comparison: we worked with hrcC concentrations of: 10^8^ CFU/ml; and flg22: 1 µM. Tsuda et al. [48] on the other hand, found comparable activation values for three out of five genes at 3–24 *hpi*, using hrcC: 5*10^7^ CFU/ml and flg22: 10 µM. That is, Tsuda et al. used lowest concentration of bacteria and highest concentration of flg22. So in general we feel that concentrations do matter, but there might be a certain time frame shift between flg22 and hrcC responses of plants as well. For clear description of this phenomenon, a high throughput assay of treatments with flg22 and hrcC mutant bacteria would be desirable with time points ranging from cca. 30 *mpi* to cca. 12 *hpi*. However, using appropriate controls, we do think that the conclusions from our and the above mentioned studies are valid.

It was interesting that genes of reactions leading to lignin formation were overrepresented while branches to salicylic acid, flavonoids, anthocyanins, and stilbenes were not present at all (Figure 4). This was not unexpected based on our [19] and others' [13] findings that salicylic acid (SA) is not an important factor of local PTI in tobacco. The innovative work of Huang et al. [49] showed the lack of SA accumulation after inoculation of tobacco with *P. syringae* pv. *tomato DC3000 hrcC^−^* mutant using a SA biosensor.

### Inhibition of C4H activity and PTI development by PIP

We applied PIP, a potent selective inhibitor of C4H – which is an early rate-limiting enzyme of the phenylpropanoid pathway – to investigate if disturbing the pathway at a key step has any effect on the outcome of PTI induction (Figure 5).

According to our experiments (Information S4) injected PIP seems to have a short inhibitory action on bacteria *in planta*, but paralelly it seems to have a longer effect on PTI development. One possible explanation of this would be that PIP, as a quasi-irreversible inhibitor could permanently bind to the active sites of the available cinnamic-acid-4-hydroxylase (C4H) enzymes, thus blocking enzymatic activity for a longer period of time. It is possible that only the excess free amount of PIP is metabolized soon afterwards. However, this explanation is hypothetic and requires further studies.

Our HR-inhibition test results indicated that a significant decrease in the HR-inhibiting ability of PTI-inducing bacteria occurred (Figure 5A, 5B). This means that inhibition of C4H by PIP inhibited PTI as well to a certain extent, determined by our experiments. Thus, disturbing the phenylpropanoid pathway at an early stage had an adverse effect on the efficiency of PTI. Visual evaluation of the HR-inhibition tests was also supported by electrolyte leakage measurements, where elevations in conductivity are related to the degree of HR necrosis.

In the context of plant-fungus interactions inhibitors of the phenylpropanoid pathway were shown to weaken barley resistance to *B. graminis*. However, genetic evidence for the phenylpropanoid pathway in cell-wall associated resistance is rare, probably because of the redundant nature of the involved enzymes and/or knock-out phenotypes often resulting in degenerated phenotypes [12].

Recently Chakravarthy et al [50] used virus-induced gene silencing (VIGS) and a cell death-based assay similar to our HR-test to assess PTI in *Nicotiana benthamiana*. Their cell death-based assay is very spectacular. Interestingly, screening a tomato library of PAMP induced genes, they found VIGS of a C4H gene to compromise PTI in their experimental system. This tomato C4H segment (GW691616) is 89% identical to the complete tobacco C4H CDS (DQ350353.1) corresponding to the C4H contig investigated in this manuscript. Their finding further supports our result obtained by enzyme inhibition. As VIGS seems a good method to test the importance of various genes in the development of PTI we also plan to carry out VIGS of the tobacco phenylpropanoid genes identified as PAMP-induced in this paper. An alternative method to assessing the role of the phenylpropanoid pathway by inhibitors would be repression or overexpression of genes controlling the pathway, such as MYB type transcription factors. Overexpression of the NtMYBGR1 transcription factor for instance caused overexpression of PAL, 4CL, C4H and OMT genes in tobacco cell culture [51].

### p-Coumarate formation is linked to PTI elicitation in tobacco

To assess, if the actual flux of phenolic compounds follows our assumptions based on HR-inhibition tests and transcriptional data, we quantified cinnamate and p-coumarate, the second and third phenolic compounds in the phenylpropanoid pathway (as cinnamic and p-coumaric acids) by the use of a TLC-densitometry method (Figure 6A and B). Transcriptional and HR-inhibition test data implied that C4H activity should be enhanced during PTI. Therefore we expected a decrease in cinnamate and a parallel increase in p-coumarate levels in PTI-induced samples. This exactly occurred in the case of treatment with PTI-inducing *P. syringae* pv. *syringae hrcC*- bacteria. However, in the case of flg22 peptide treatment only the expected increase in p-coumarate level was observed, while the level of cinnamic acid did not change in these 6 *hpi* samples as compared to the DMSO control. A possible explanation to this phenomenon could be that a dynamic equilibrium state of the cinnamate level was maintained here, which would mean that the PAL enzyme could keep up production of cinnamate from phenylalanine at the rate of C4H converting cinnamate to p-coumarate. In the case of *P. syringae hrcC*- bacteria a higher level of C4H induction might have occurred, with which PAL could not keep up completely, therefore a relative decrease in cinnamate occurred. We do not know however if there are other enzymes involved in converting cinnamate into different derivatives, for example into salicylic acid. Therefore the exact enzymatic mechanisms creating the measured changes in the two phenolic acids will require more studies, for example a higher scale metabolic profiling experiment, preferably including a time series with earlier time points than the 6 *hpi* samples used here, and/or also applying higher concentrations of flg22.

The basic level of cinnamate increased when PIP was applied. This could mean that a flow of cinnamate towards another compound(s) is blocked by PIP, therefore cinnamate accumulated. We know that PIP is a specific inhibitor of C4H therefore we expected this result [32]. Also, coumarate levels in PTI-induced samples were reduced when PIP was co-infiltrated with either of the two used elicitors. We can say that these TLC derived data correlated well with data from HR-inhibition experiments (Figure 5).

Real time RT-PCR investigation of activity changes of the identified phenylpropanoid genes in response to *P. s. syringae hrcC*-, flg22 and their combinations with PIP (Figure 6C) showed elevation of gene activities of certain genes in the PIP-combination treatments, as compared to the corresponding elicitor alone-treatment. These genes were exactly the first five investigated PPP genes (PALa, PALb, C4H, 4CL, OMT-I), closest to the origin of the pathway. This could be caused by a compensation feedback present in the plant leaves, which senses lack of C4H products in some way. The first four investigated PPP genes (PALa, PALb, C4H, 4CL) were slightly activated upon treatment solely with PIP, and of the four, activation of C4H was highest, about 3-fold. This might again be result of a feedback loop sensing C4H activity itself, or p-coumarate, or any downstream products. The actual mechanisms behind these findings require further investigations.

All in all, what our results strongly imply is that cinnamate is at least partly converted into p-coumarate during the course of PTI. This even seems to correlate with the strength of PTI, based on our HR-inhibition tests and electrolyte-leakage experiments.

A lot of questions arise or remain to answer however. For example, looking at the absolute value of the decrease in cinnamate and increase in p-coumarate we see that they are not identical. The difference between *P. s. hrcC*- and PIP+*P. s. hrcC*- samples is about +17 nmol/g fresh weight in the case of cinnamate, while only -2.1 nmol/g fresh weight with coumarate. Based on these data we cannot say that “the exact amount that was not converted to p-coumarate stayed cinnamate”. This is not surprising however, as we are dealing with a whole network of interconnected enzymatic reactions, phenolic compounds can go into insoluble fraction from soluble fraction, the amount of the enzymes themselves also changes as their mRNA levels change and even measurements might be biased.

Based on transcriptional activation, HR-inhibition tests, electrolyte leakage experiments, and TLC results we concluded that C4H seems to play an important role in the development of PTI. This however raises a lot of further questions to be answered in the future. We are planning to develop a HPLC based method to measure changes of phenolic compounds at a higher scale, to better understand the roles of these compounds in PTI. We are especially curious about the changes in the levels of phenylalanine, salicylic acid, other phenolic acids and alcohols, flavonoids etc. We are also planning VIGS of the phenylpropanoid genes investigated here, to test the effect of their inhibition on the development of PTI.

Investigating the role of PPP in PTI, one shall remember that the presumably formed phenolic acids (i.e. coumarate, caffeate, ferulate) have been shown to have direct antimicrobial activity [52], which might also add to the efficiency of PTI. Importance of the antioxidant activity of phenylpropanoid products has been shown by Kostyn et al. [53] in the plant–fungus interaction context. The detected transcriptional activation of flax phenylpropanoid genes in response to *Fusarium* infection was supported by metabolite profiling. Elevated antioxidant status of infected plants was verified by measuring ROS (Reactive Oxygen Species) scavenging activities of identified compounds.

The true role of phenylpropanoid compounds in PTI against bacteria still remains to establish. Matching metabolic profiles to transcriptional changes are among our future plans as well as assessing antibacterial activities and antioxidant capacities of the to-be-identified phenolic molecules.

## Supporting Information

Information S1 **Contigs identified from tobacco PTI (Pattern Triggered Immunity).** Tobacco leaves were infiltrated with Pseudomonas syringae pv. syringae hrcC- bacteria. Samples were taken 6 and 48 hpi. Suppression Subtractive Hybridization was carried out to build a clone library of PTI-related transcripts. Sequencing and sequence assembly yielded 176 contigs described in the table below.
(PDF)

Information S2 **Visualization of changes in expression during PTI response of tobacco as represented by the Biotic Stress Overview in MapMan.** A) 6 hpi. B) 48 hpi. Mean expression ratio of the PTI-related activated contigs from our tobacco cDNA microarray in PTI-induced vs. water treated samples was calculated based on three replicate experiments per time point and log-scaled data were visualized using the MapMan “BioticStress” pathway (http://mapman.gabipd.org). Individual transcripts are symbolized by red or white boxes. The scale bar represents fold change and reaches to +2.5 (deep red) on the log scale, which corresponds to a 5.7 fold change in the linear scale.
(PDF)

Information S3 **Verification of gene-activation by real-time RT-PCR: data corresponding to** **Fig. 3** **.** Ratios of mRNA levels are compared to water-treated controls. Tobacco leaves were infiltrated with P. syringae pv. syringae hrcC- suspension or flagellin22 peptide, samples were taken 3, 6 and 48 hours later. Flagellin-treated leaves were sampled earlier, because of earlier timing of gene activation. Values are averages of three independent biological replicates. Each replicate was normalized by corresponding actin levels. Stars indicate significant gene activation (p<0.05) as compared to water treated controls.
(PDF)

Information S4 **Representative images showing the effect of PIP pre-treatment; and live versus heat-killed** ***P. syringae hrcC*** **pre-treatments on the HR induced by** ***Pseudomonas syringae*** **pv.** ***syringae*** **61.** A) Effect of PIP and DMSO on HR-inducing activity of *Pseudomonas syringae* pv. *syringae* 61 as a function of time elapsed after the pre-treatment. B) Representative image of the effect of live and heat-killed *P. syringae hrcC* pre-treatments combined with PIP on the extension of the HR lesion induced by *P. syringae* 61 challenge inoculation.
(PDF)

Information S5 **Primer sequences used for tobacco real-time RT-PCR in this work.** The primers in the list were designed for *Nicotiana tabacum* cv. *Samsun* - derived gene fragments to assess expressional activation of the genes during PTI. The primers anneal optimally at 60°C and result in a PCR product of 50-150 basepairs. Therefore these primers can be run in parallel PCR reactions among the same conditions, without further optimization.
(PDF)
