# Supplementary material for: Pattern Triggered Immunity (PTI) in Tobacco: Isolation of Activated Genes Suggests Role of the Phenylpropanoid Pathway in Inhibition of Bacterial Pathogens
Source: PLoS One. 2014 Aug 7;9(8):e102869. doi: 10.1371/journal.pone.0102869 (PMC4125134; doi:10.1371/journal.pone.0102869)
Supplement: Information S1 — Contigs identified from tobacco PTI (Pattern Triggered Immunity). Tobacco leaves were infiltrated with Pseudomonas syringae pv. syringae hrcC- bacteria. Samples were taken 6 and 48 hpi. Suppression Subtractive Hybridization was carried out to build a clone library of PTI-related transcripts. Sequencing and sequence assembly yielded 176 contigs described in the table below. (PDF) [file pone.0102869.s001.pdf]

Supporting Information S1. Contigs identified from tobacco PTI (Pattern Triggered Immunity). Tobacco leaves were infiltrated with *Pseudomonas syringae* pv. *syringae hrcC-* bacteria. Samples were taken 6 and 48 *hpi* . Suppression Subtractive Hybridization was carried out to build a clone library of PTI-related transcripts. Sequencing and sequence assembly yielded 176 contigs described in the table below.

Contig number	Length (base-pairs)	Number of clones in contig	Genbank accession	Gene name	Blast "expect" value	Identity
**CELL STRUCTURE**						
13	749	3	AY422690	N. attenuata tissue-type shoot beta-tubulin (TUB)	0	598/612 (97%)
156	333	1	AJ421413	N. tabacum mRNA for alpha-tubulin (tubA3 gene)	0	325/325 (100%)
**CELL PROTECTION**						
8	519	9	X78203	H.muticus mRNA for glutathione S-transferase	7e-92	276/310 (89%)
52	352	2	U57350	N. tabacum epoxide hydrolase	e-179	344/352 (97%
86	495	1	Y12013	D. carota mRNA for RAD23 protein, isoform I	1e-25	111/127 (87%)
98	466	1	AY383545	A. thaliana NADK1 (At3g21070)	2,00E-14	77/88 (87%)
102	348	1	X55974	N.plumbaginifolia mRNA for superoxide dismutase	e-123	307/331 (92%)
131	228	1	U57350	N. tabacum epoxide hydrolase	e-116	223/226 (98%)
155	627	1	DQ088821	G. hirsutum FeSOD mRNA	2,00E-21	232/290 (80%)
**CELLWALL**						
2	613	26	AB041519	N. tabacum mRNA for P-rich protein, NtEIG-C29	0	474/514 (92%)
3	539	13	Y19032	N. tabacum cell wall protein (TLRP tyrosine- and lysine-rich protein)	e-170	366/396 (92%)
4	284	6	AF151215	N. glauca cell-type guard cell glycine-rich protein	2e-07	41/44 (93%)
5	766	16	AB035125	N. tabacum NtEIG-E17 for glycine-rich protein	0	535/536 (99%)
21	372	4	X70343	N.sylvestris mRNA for extensin	e-176	350/370 (94%)
22	144	1	AJ271872	N. sylvestris Ext1.2B gene for extensin	9,00E-70	142/144 (98%)
31	168	4	D86721	N. tabacum for glycine-rich protein	8,00E-89	168/168 (100%)
42	500	2	AB041517	N. tabacum NtEIG-A1 mRNA	e-131	243/244 (99%)
44	189	3	AB041516	N. tabacum mRNA for P-rich protein EIG-I30	e-100	188/188 (100%
74	298	1	AF291179	C. annuum stellacyanin-like protein CASLP1 precursor	6,00E-54	182/206 (88%)
75	387	1	AB041517	N. tabacum NtEIG-A1	e-105	227/237 (95%)
**DEFENCE-RELATED**						
6	789	16	AB044153	N. tabacum mRNA for peroxidase	0	761/783 (97%)
9	454	6	X83851	N. tabacum mRNA for PAR-1b	0	452/454 (99%)
10	223	3	AB041515	N. tabacum NtEIG-E80 mRNA, complete cds	1e-47	187/217 (86%)
11	293	8	M97360	N. tabacum protein SAR8.2c	e-160	288/288 (100%)
19	354	5	AB267862.1	N. tabacum NtChitIV gene for class IV chitinase	3,00E-158	339/355 (95%)
26	462	4	AJ309300	S. tuberosum putative membrane protein	e-121	355/399 (88%)
29	176	4	L02124	N. tabacum anionic peroxidase gene	2,00E-92	174/174 (100%)
71	507	2	Z11564	N. tabacum mRNA for basic chitinase III	0	507/508 (99%)
72	106	2	AJ810538	N. tabacum partial tpoxN1 gene for peroxidase N1	3e-51	105/106 (99%)
87	225	1	BT012770	L. esculentum clone 113748F	6e-75	202/221 (91%)

99	156	1	AJ309384	A. belladonna putative NtPRp27-like protein	5,00E-25	73/77 (94%)
109	210	1	AY562133	N. tabacum TCIP mRNA	5,00E-66	191/210 (90%)
118	314	1	X81834	N. tabacum mRNA for beta-fructosidase	e-171	312/314 (99%)
121	241	1	AY237117	C. annuum leucine-rich repeat protein (LRR1)	2,00E-47	201/235 (85%)
134	244	1	X12737	N. tabacum PR-1a pathogenesis-related protein	e-134	244/244 (100%)
140	293	1	AF154636	N. tabacum clone PR5 sequence	e-143	262/263 (99%)
**HEAT SHOCK PROTEINS**						
14	624	1	Z71395	N.plumbaginifolia calreticulin	6,00E-59	125/127 (98%)
28	917	4	L08830	L. esculentum BiP (binding protein)/grp78 (glucose-regulated protein, 78 kD)	0	791/914 (86%)
35	337	3	AJ538960	N. tabacum cDNA-AFLP-fragment	2,00E-14	62/68 (91%)
38	247	3	AY368274	N. tabacum cyclophilin-like (CYP1) mRNA	e-125	242/246 (98%)
47	731	2	X60058	N. tabacum blp5 luminal binding protein (BiP)	0	721/723 (99%)
111	396	1	BT004527	A. thaliana At4g24190 HSP90-like protein	2e-39	179/210 (85%)
133	400	1	L14594	C. roseus heat shock protein 90	5,00E-77	308/361 (85%)
145	257	1	Z71395	N.plumbaginifolia calreticulin	7,00E-72	146/148 (98%)
153	77	1	AY253832	L. esculentum copper chaperone (LeCCH)	2,00E-13	53/57 (92%)
**ENERGY, METABOLISM**						
12	320	7	AF082893	S. tuberosum methionine synthase (MS)	e-101	239/255 (93%)
18	579	5	AF127796	C. chinense acyl carrier protein (Acl1)	6,00E-99	267/294 (90%)
20	306	5	AB061256	S. tuberosum NADP-dependent malic enzyme	e-116	275/295 (93%)
32	154	4	AF001270	L. esculentum cytosolic NADP-malic enzyme 2	8,00E-61	145/153 (94%)
34	479	3	AF307144	S. oleracea cytosolic 6-phosphogluconate dehydrogenase (pgdC)	7,00E-24	204/252 (80%)
36	408	3	AF321140	N. tabacum S-adenosylmethionine synthase	0	408/408 (100%)
41	144	3	AF321140	N. tabacum S-adenosylmethionine synthase	2,00E-73	142/142 (100%)
46	145	3	X71441	N. tabacum cytochrome b5	2,00E-42	129/142 (90%)
60	277	2	X58108	L. esculentum mRNA for enolase	e-112	259/277 (93%)
69	107	2	AF024634	P. crispum NADPH cytochrome P450 reductase	7e-08	41/44 (93%)
77	417	1	AF082893	S. tuberosum methionine synthase (MS)	e-151	381/417 (91%)
80	216	1	D16138	N. tabacum cytokinin binding protein CBP57 (S-adenosyl-L-homocystein hydrolase)	e-115	215/216 (99%)
92	102	1	D16139	N. tabacum cytokinin binding protein CBP57	3,00E-47	101/102 (99%)
103	413	1	AY224189	M. truncatula adenosylhomocysteinase (AHC2)	2,00E-57	260/308 (84%)
105	496	1	BT020249	A. thaliana At2g30720 thioesterase family protein	4,00E-19	79/88 (89%)
115	333	1	X82325	S. tuberosum cytochrome c reductase	7e-11	131/162 (80%)
122	492	1	AF001270	L. esculentum cytosolic NADP-malic enzyme	e-164	443/492 (90%)
126	286	1	AB007907	G. max 6-phosphogluconate dehydrogenase	3,00E-31	135/156 (86%)
162	261	1	AY695053.	N. tabacum adenosine kinase isoform 2S	e-144	261/261 (100%)
170	175	1	AF001270.	L. esculentum cytosolic NADP-malic enzyme	1,00E-63	159/170 (93%)
174	188	1	AF082893.	S. tuberosum methionine synthase (MS)	9,00E-12	63/71 (88%)
**MISCELLANEOUS**						
7	195	10	AB206920.1	C. annuum catf2 gene for acyl-transferase	7e-14	69/78 (88%)
17	292	6	BT014186	L. esculentum clone 133363F	5,00E-42	141/158 (89%)
30	949	4	AY819026	C. frutescens acyltransferase (Pun1)	7,00E-91	301/346 (86%)
63	211	2	D26460	N. glauca X N. langsdorffii tumor-related protein	1e-11	39/39 (100%)
91	333	1	BT009240	T. aestivum clone wlk1.pk0007.f5:fis	2e-14	128/156 (82%)
132	480	1	AF243180	L. esculentum dicyanin	8,00E-64	249/289 (86%)
147	218	1	D26106	C. sativus ferrochelatase	1,00E-11	105/127 (82%)
**PHENYLPROPANOID**						
1	449	39	X74453	N. tabacum OMT I-b mRNA	0	438/447 (97%)

39	654	3	AF150881	L. esculentum x L. peruvianum ferulate-5-hydroxylase (CYP84)	4,00E-23	167/203 (82%)
45	172	3	AF212318	C. annuum cinnamic acid 4-hydroxylase (C4H)	3e-14	103/123 (83%)
51	468	2	U50845	N. tabacum 4-coumarate:Co-A ligase (4CL)	0	466/468 (99%)
64	209	2	D17467	N. tabacumphenylalanine ammonia-lyase	e-111	208/209 (99%)
84	652	1	X78269	N. tabacum phenylalanine ammonia-lyase	0	650/652 (99%)
136	137	1	X74452	N .tabacum OMT I-a mRNA	5,00E-68	136/137 (99%)
165	649	1	Z21796	L. esculentum chorismate synthase 1 precursor	0	458/494 (92%),
175	498	1	X71430	N. tabacum O-diphenol-O-methyltransferase.	0	497/498 (99%)
**PHOTOSYNTHESIS**						
104	219	1	X17578	S. tuberosum 33kDa precursor protein of the oxygen-evolving complex	5e-32	145/167 (86%)
169	329	1	AB012636.	N. sylvestris Lhcb1*1 gene for light harvesting chlorophyll a/b-binding protein	e-153	316/329 (96%)
**PROTEIN METABOLISM**						
25	484	1	AB177386	D. caryophyllus DC-CP2 cysteine proteinase	4,00E-04	60/71 (84%)
56	270	2	DQ016993	I. batatas putative L24 ribosomal protein	6e-57	212/245 (86%
62	219	2	BT014015	L. esculentum clone 133077F ribosomal protein	5,00E-23	86/94 (91%)
65	195	2	AY504966	I. hollandica putative cysteine protease 1(CYS1)	0,002	37/41 (90%)
97	248	1	AF332451	A. thaliana putative E2, ubiquitin-conjugating enzyme 1 (At1g14400)	3,00E-34	131/149 (87%)
112	258	1	Z31720	N. tabacum L19 ribosomal protein L19	2e-78	223/247 (90%)
120	90	1	Y09505	N. tabacum mRNA for delta proteasome subunit	5,00E-33	86/90 (95%)
127	140	1	AF370549	A. thaliana Putative ribosomal protein (F5A13.4)	0,006	
159	279	1	AY662988	P. hybrida cysteine proteinase (CP2)	2,00E-72	204/225 (90%)
161	163	1	AJ844617	P. major mRNA for polyubiquitin (ubq3 gene).	1,00E-41	143/161 (88%)
163	414	1	AK121755	O. sativa (japonica cultivar-group) cDNA	4,00E-22	69/73 (94%)
167	387	1	AY133535	A. thaliana At5g60160/f15l12_20	3,00E-19	175/216 (81%)
171	429	1	AJ223329	N. tabacum TUQG3 gene, complete CDS.	0	410/429 (95%)
**SIGNALLING**						
24	409	3	AB117525	N. tabacum NtMKP1 mRNA for MAP kinase	0	335/335 (100%)
53	375	2	AY904339	C. annuum calcium-dependent protein kinase 4 (CDPK4)	1e-15	73/82 (89%)
54	332	2	Z29529	N. tabacum ethylene forming enzyme (EFE).	0	331/332 (99%)
55	292	2	U91723	N. tabacum 14-3-3 isoform b T14-3b	e-162	292/292 (100%)
58	253	2	AK221707	A. thaliana MAP3K-like protein kinase	1,00E-17	91/105 (86%)
59	271	2	D26015	N. tabacum CND41, chloroplast nucleoid DNA binding protein with aspartic protease activity	e-136	262/269 (97%)
66	169	2	L29275	N. tabacum SR1 Nt-rab7b	1e-87	166/166 (100%)
90	140	1	AB073628	N. tabacum mRNA for receptor-like protein kinase	3e-72	140/140 (100%)
94	280	1	AY673975	S. tuberosum phosphatidylinositol synthase	e-113	260/277 (93%)
95	190	1	AF211529	N. tabacum Avr9/Cf-9 rapidly elicited protein 31 (ACRE31)	e-102	190/190 (100%)
106	451	1	AJ276267	C. arietinum mRNA for putative PTS protein	1e-22	205/254 (80%)
108	387	1	Z29529	N. tabacum ethylene forming enzyme (EFE)	0	386/387 (99%)
124	125	1	U91723	N. tabacum 14-3-3 isoform b T14-3b	3,00E-63	125/125 (100%)
125	637	1	Y11348	M.sativa annexin-like protein	8e-09	107/131 (81%)
135	354	1	AF478458	C. annuum DNA binding protein Rav	7,00E-82	271/309 (87%)
137	129	1	AB073628	N. tabacum mRNA for receptor-like protein kinase	3,00E-35	115/126 (91%)
139	375	1	AY972084	O. sativa sphingosine-1-phosphate lyase (SPL)	2,00E-17	70/77 (90%)
142	361	1	AY227667	L. esculentum myo inositol monophosphatase isoform 2 (IMP-2)	1,00E-21	89/100 (89%)
144	450	1	U94748	P. hybrida WD repeat protein AN11 (An11)	e-140	399/447 (89%)

151	236	1	AB014483	N. tabacum NtHSF1 heat shock factor,	e-124	234/236 (99%)
**TERPENOIDS**						
73	231	2	AF542544	N. attenuata 5-epi-aristolochene synthase	e-104	207/226 (91%)
130	116	1	AF004233	N. tabacum hydroxy-methyl-glutaryl-coenzyme A reductase (HMGRL)	9,00E-54	116/117 (99%)
164	185	1	AY497303	N. benthamiana (E)-4-hydroxy-3-methylbut-2-enyl diphosphate synthase	4,00E-94	183/185 (98%)
173	460	1	BT013122.	L. esculentum mevalonate disphosphate decarboxylase	0	430/460 (93%
**TRANSPORT**						
15	604	1	BT013651	L. esculentum putative ABC transporter	e-145	330/352 (93%)
16	409	1	AF347614	L. esculentum sulfate transporter 2 mRNA	e-131	362/403 (89%)
23	407	4	BT013251	L. esculentum putative ABC transporter	e-116	359/407 (88%)
57	266	2	AF290618	N. glauca putative delta TIP (MIP2)	3e-49	117/122 (95%)
67	156	2	X66856	N. tabacum MST1 mRNA	1e-81	156/156 (100%)
89	714	1	BT014249	L. esculentum clone putative coatomer protein	e-175	533/606 (87%)
101	503	1	AY220478	N. tabacum Avr9/Cf-9 rapidly elicited protein 141 (ACRE141)	e-172	454/503 (90%)
107	142	1	AB075550	N. tabacum NtPDR1 mRNA for pleiotropic drug resistance like protein	5e-71	141/142 (99%)
138	119	1	X62123	S. tuberosum ant gene for ADP/ATP translocator	8,00E-48	114/119 (95%)
143	318	1	AJ563367	G. max sorbitol-like transporter (stp gene).	6,00E-11	98/118 (83%)
154	144	1	AY302065.	P. tomentosa ADP-ribosylation factor 1	2,00E-55	136/144 (94%)
172	397	1	U64823	N. sylvestris amino acid permease (nsaap1)	1,00E-74	235/265 (88%)
**UNKNOWN**						
27	310	4	BT014512	L. esculentum clone	1,00E-30	197/239 (82%)
40	251	3	AJ00321	S. tuberosum mRNA for hypothetical protein	1,00E-30	200/243 (82%)
68	245	2	AY087786	A. thaliana clone 38412 mRNA	2e-07	59/68 (86%)
79	469	1	AC158209	M. truncatula clone mth2-154h5	1e-09	81/96 (84%)
83	440	1	AJ718559	N. tabacum cDNA-AFLP-fragment BSTC2-32-400	e-178	334/339 (98%)
88	573	1	AP004525	L. corniculatus var. japonicus genomic DNA	0,007	46/53 (86%)
100	392	1	BT013096	L. esculentum clone 114362R	e-174	372/392 (94%)
114	128	1	BT014442	L. esculentum clone 133764F	8e-39	117/128 (91%)
148	485	1	AP008211	O. sativa genomic DNA, chromosome 5.	2,00E-08	55/62 (88%)
150	362	1	AL161595.2	A. thaliana DNA chromosome 4, con. fragment 91.	1	36/41 (87%)
166	341	1	AC155101.	M. truncatula clone mth2-139j13	3,00E-07	86/104 (82%)
**NO SIGNIFICANT SIMILARITY**						
33	39	4	NA			
37	255	3	NA			
43	255	1	NA			
48	598	2	NA			
49	550	2	NA			
50	499	2	NA			
61	220	2	NA			
70	173	2	NA			
76	30	2	NA			
78	145	1	NA			
81	378	1	NA			
82	368	1	NA			
85	115	1	NA			
93	248	1	NA			

96	275	1	NA
110	167	1	NA
113	451	1	NA
116	185	1	NA
117	633	1	NA
119	80	1	NA
123	297	1	NA
128	346	1	NA
129	399	1	NA
141	169	1	NA
146	30	1	NA
149	300	1	NA
152	271	1	NA
157	128	1	NA
158	171	1	NA
160	177	1	NA
168	267	1	NA
176	9	1	NA
