# Supplementary material for: Pattern Triggered Immunity (PTI) in Tobacco: Isolation of Activated Genes Suggests Role of the Phenylpropanoid Pathway in Inhibition of Bacterial Pathogens
Source: PLoS One. 2014 Aug 7;9(8):e102869. doi: 10.1371/journal.pone.0102869 (PMC4125134; doi:10.1371/journal.pone.0102869)
Supplement: Information S2 — Visualization of changes in expression during PTI response of tobacco as represented by the Biotic Stress Overview in MapMan. A) 6 hpi. B) 48 hpi. Mean expression ratio of the PTI-related activated contigs from our tobacco cDNA microarray in PTI-induced vs. water treated samples was calculated based on three replicate experiments per time point and log-scaled data were visualized using the MapMan “BioticStress” pathway (http://mapman.gabipd.org). Individual transcripts are symbolized by red or white boxes. The scale bar represents fold change and reaches to +2.5 (deep red) on the log scale, which corresponds to a 5.7 fold change in the linear scale. (PDF) [file pone.0102869.s002.pdf]

Supporting Information S2. Visualization of changes in expression during PTI response of tobacco as represented by the Biotic Stress Overview in MapMan. A) *6 hpi*. B) *48 hpi*.

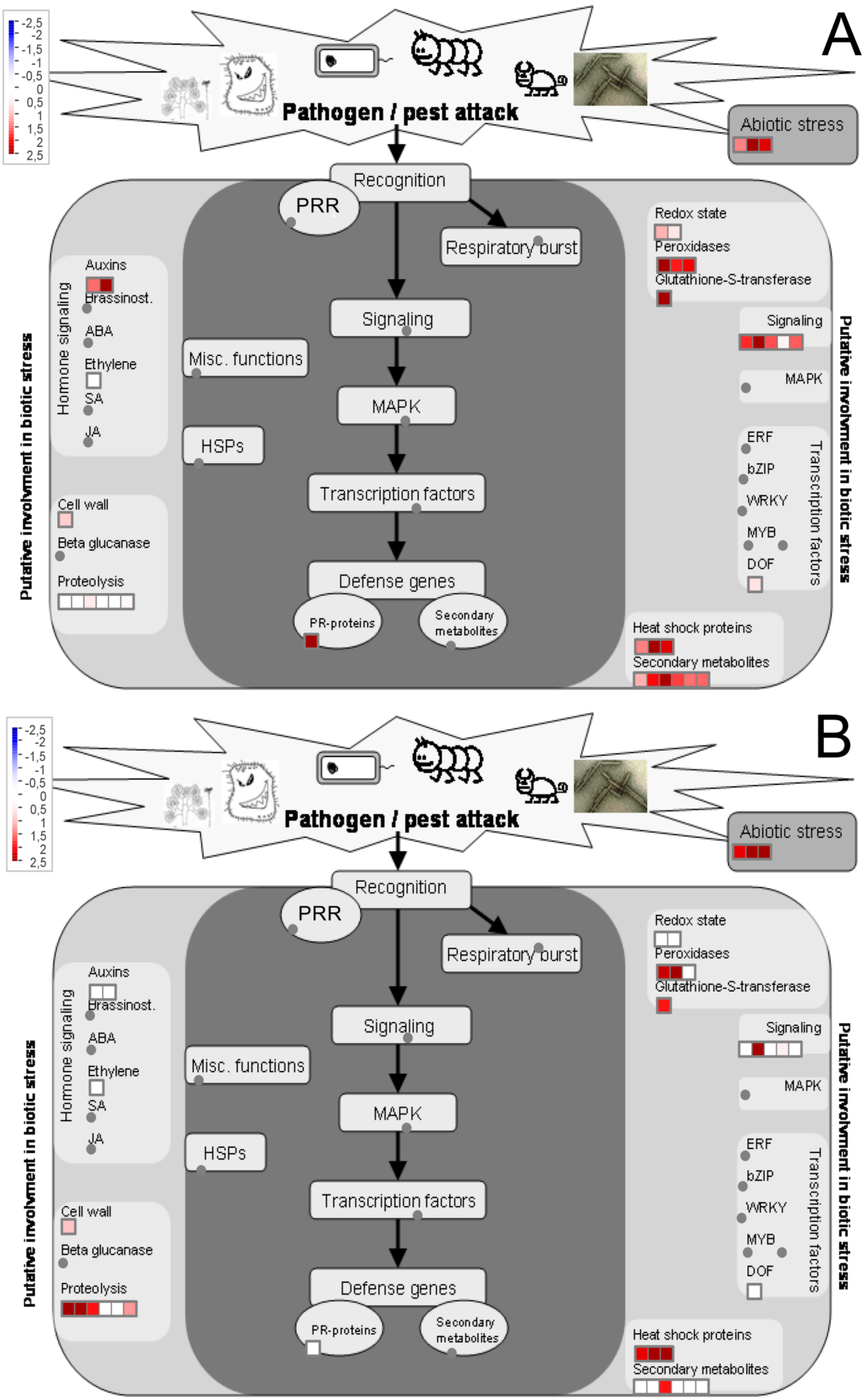

Mean expression ratio of the PTI-related activated contigs from our tobacco cDNA microarray in PTI-induced vs. water treated samples was calculated based on three replicate experiments per time point and log-scaled data were visualized using the MapMan “BioticStress” pathway (http://mapman.gabipd.org).

Individual transcripts are symbolized by red or white boxes. The scale bar represents fold change and reaches to +2.5 (deep red) on the log scale, which corresponds to a 5.7 fold change in the linear scale.
