# Supplementary material for: Pattern Triggered Immunity (PTI) in Tobacco: Isolation of Activated Genes Suggests Role of the Phenylpropanoid Pathway in Inhibition of Bacterial Pathogens
Source: PLoS One. 2014 Aug 7;9(8):e102869. doi: 10.1371/journal.pone.0102869 (PMC4125134; doi:10.1371/journal.pone.0102869)
Supplement: Information S3 — Verification of gene-activation by real-time RT-PCR: data corresponding to Fig. 3 . Ratios of mRNA levels are compared to water-treated controls. Tobacco leaves were infiltrated with P. syringae pv. syringae hrcC- suspension or flagellin22 peptide, samples were taken 3, 6 and 48 hours later. Flagellin-treated leaves were sampled earlier, because of earlier timing of gene activation. Values are averages of three independent biological replicates. Each replicate was normalized by corresponding actin levels. Stars indicate significant gene activation (p<0.05) as compared to water treated controls. (PDF) [file pone.0102869.s003.pdf]

Supporting Information S3. Verification of gene-activation by real-time RT-PCR: data corresponding to Fig. 3.

Ratios of mRNA levels are compared to water-treated controls. Tobacco leaves were infiltrated with *P. syringae* pv. *syringae hrcC-* suspension or flagellin22 peptide, samples were taken 3, 6 and 48 hours later. Flagellin-treated leaves were sampled earlier, because of earlier timing of gene activation. Values are averages of three independent biological replicates. Each replicate was normalized by corresponding actin levels. Stars indicate significant gene activation (p<0.05) as compared to water treated controls.

functional group	contig number	gene name	treatment	3 hpi fold	3 hpi sd	6 hpi fold	6 hpi sd	48 hpi fold	48 hpi sd
Signal transduction-related genes	C171	UBIE (ubiquitin extension protein)	PS hrcC	-	-	1,30	0,48	1,44	0,51
	C139	sphingosine-1-phosphate lyase (SPL)		-	-	0,61	0,11	1,04	0,08
	C66	SR1 Nt-rab7b membrane-associated GTP-binding		-	-	0,89	0,20	1,31	0,18
	C59	CND41, chloroplast nucleoid DNA binding protein		-	-	1,07	0,20	5,56	5,30
	C94	phosphatidylinositol synthase		-	-	1,33	0,16	1,41	0,35
	C106	putative PTS HPR protein (serine-phosphorilation)		-	-	1,45	0,66	3,72 *	1,54
	C24	NtMKP1 MAP kinase		-	-	1,53	0,39	2,89 *	0,65
	C90	receptor-like protein kinase		-	-	1,61	0,47	2,28 *	0,65
	C108	ethylene forming enzyme (EFE)		-	-	2,06	1,19	2,77 *	1,28
	C125	annexin-like protein		-	-	2,25	0,53	5,40 *	2,44
	C144	WD repeat protein AN11 (An11)		-	-	2,97 *	1,22	1,88	1,07
	C55	14-3-3 protein isoform b T14-3b		-	-	3,04	2,59	1,54	0,60
	C135	DNA binding protein Rav		-	-	3,58 *	1,05	3,25 *	0,81
	C53	calcium-dependent protein kinase 4 (CDPK4)		-	-	3,85 *	0,85	2,74	1,23
	C58	MAP3K-like protein kinase		-	-	4,12 *	1,76	1,76	0,58
	C151	NtHSF1 heat shock factor,		-	-	7,03 *	3,21	2,64 *	1,87
	C95	Avr9/Cf-9 rapidly elicited protein 31 (ACRE31)		-	-	9,28 *	5,37	8,83 *	6,94
Phenylpropanoid pathway and lignification	C171	UBIE (ubiquitin extension protein)		-	-	1,30	0,48	1,44	0,51
	C51	4CL (4-hydroxycinnamoyl-CoA ligase)		-	-	5,84 *	1,94	5,65 *	2,45
	C45	C4H (cinnamate 4-hydroxylase)		-	-	6,63 *	2,60	3,91 *	2,27
	C84	PALb (Phenylalanine ammonia-lyase)		-	-	7,59 *	3,80	8,74 *	3,28
	C64	PALa (Phenylalanine ammonia-lyase)		-	-	8,27 *	5,73	9,45 *	4,12
	C72	POX (peroxidase)		-	-	12,47 *	4,68	8,60 *	4,56
	C1	OMT I (O-methyltransferase)		-	-	14,40 *	8,81	3,38 *	1,93
	C39	F5H (ferulate 5-hydroxylase)		-	-	97,97 *	59,54	21,34 *	13,89
	C51	4CL (4-hydroxycinnamoyl-CoA ligase)	flg22	3,49 *	0,22	1,09	0,18	-	-
	C45	C4H (cinnamate 4-hydroxylase)		3,86 *	0,21	0,71	0,36	-	-
	C84	PALb (Phenylalanine ammonia-lyase)		2,68 *	0,10	1,40	0,84	-	-
	C64	PALa (Phenylalanine ammonia-lyase)		5,44 *	0,47	2,00	0,41	-	-
	C72	POX (peroxidase)		4,72 *	0,49	6,94 *	0,50	-	-
	C1	OMT I (O-methyltransferase)		3,58 *	0,01	3,57	1,85	-	-
	C39	F5H (ferulate 5-hydroxylase)		18,04 *	0,87	17,93 *	1,33	-	-

*** p<0.05**

PS hrcC: Pseudomonas syringae pv. syringae hrcC-

flg22: flagelline 22 peptide
