# Supplementary material for: Pattern Triggered Immunity (PTI) in Tobacco: Isolation of Activated Genes Suggests Role of the Phenylpropanoid Pathway in Inhibition of Bacterial Pathogens
Source: PLoS One. 2014 Aug 7;9(8):e102869. doi: 10.1371/journal.pone.0102869 (PMC4125134; doi:10.1371/journal.pone.0102869)
Supplement: Information S4 — Representative images showing the effect of PIP pre-treatment; and live versus heat-killed P. syringae hrcC pre-treatments on the HR induced by Pseudomonas syringae pv. syringae 61. A) Effect of PIP and DMSO on HR-inducing activity of Pseudomonas syringae pv. syringae 61 as a function of time elapsed after the pre-treatment. B) Representative image of the effect of live and heat-killed P. syringae hrcC pre-treatments combined with PIP on the extension of the HR lesion induced by P. syringae 61 challenge inoculation. (PDF) [file pone.0102869.s004.pdf]

Supporting Information S4. Representative images showing the effect of PIP pre-treatment; and live versus heat-killed *P. syringae hrcC* pre-treatments on the HR induced by *Pseudomonas syringae* pv. *syringae* 61.

A) **Effect of PIP and DMSO on HR-inducing activity of *Pseudomonas syringae* pv. *syringae* 61 as a function of time elapsed after the pre-treatment.**

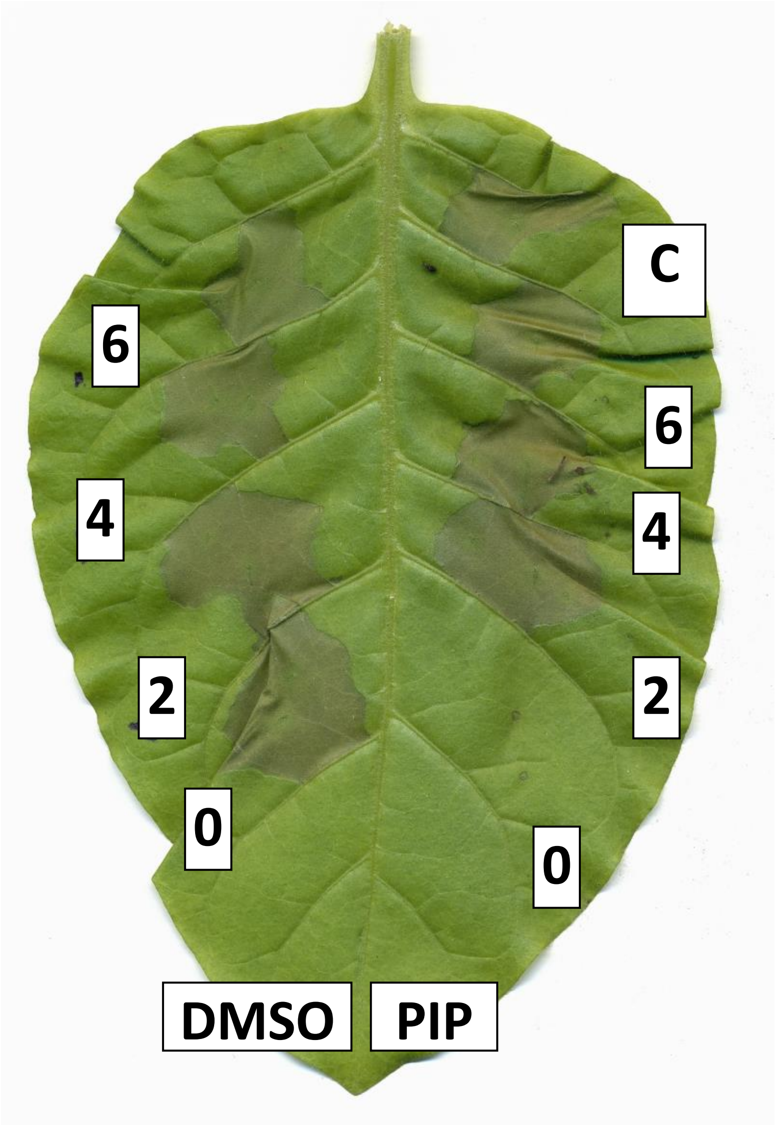

Interveinals on the left side of tobacco leaves were injected with DMSO (200X dilution) as controls and interveinals on the right side of the leaves were injected with 1mM PIP (diluted from 200 mM PIP in DMSO stock). At different time points (0, 2, 4, 6 hours) after these pre-treatments *P. syringae* pv. *syringae* 61 bacteria (10^8 CFU/ml suspension) were injected into the intervenials. 0 *hpi* treatment was carried out by combining the bacterial suspension with PIP or DMSO, and this mixture was injected immediately into plant leaves. Development of HR was observed and photo was taken one day after inoculation.

C (absolute control): 10^8 CFU/ml suspension of *P. syringae* pv. *syringae* 61 bacteria in water without PIP or DMSO.

B) **Representative image of the effect of live and heat-killed *P. syringae hrcC* pre-treatments combined with PIP on the extension of the HR lesion induced by *P. syringae 61* challenge inoculation**

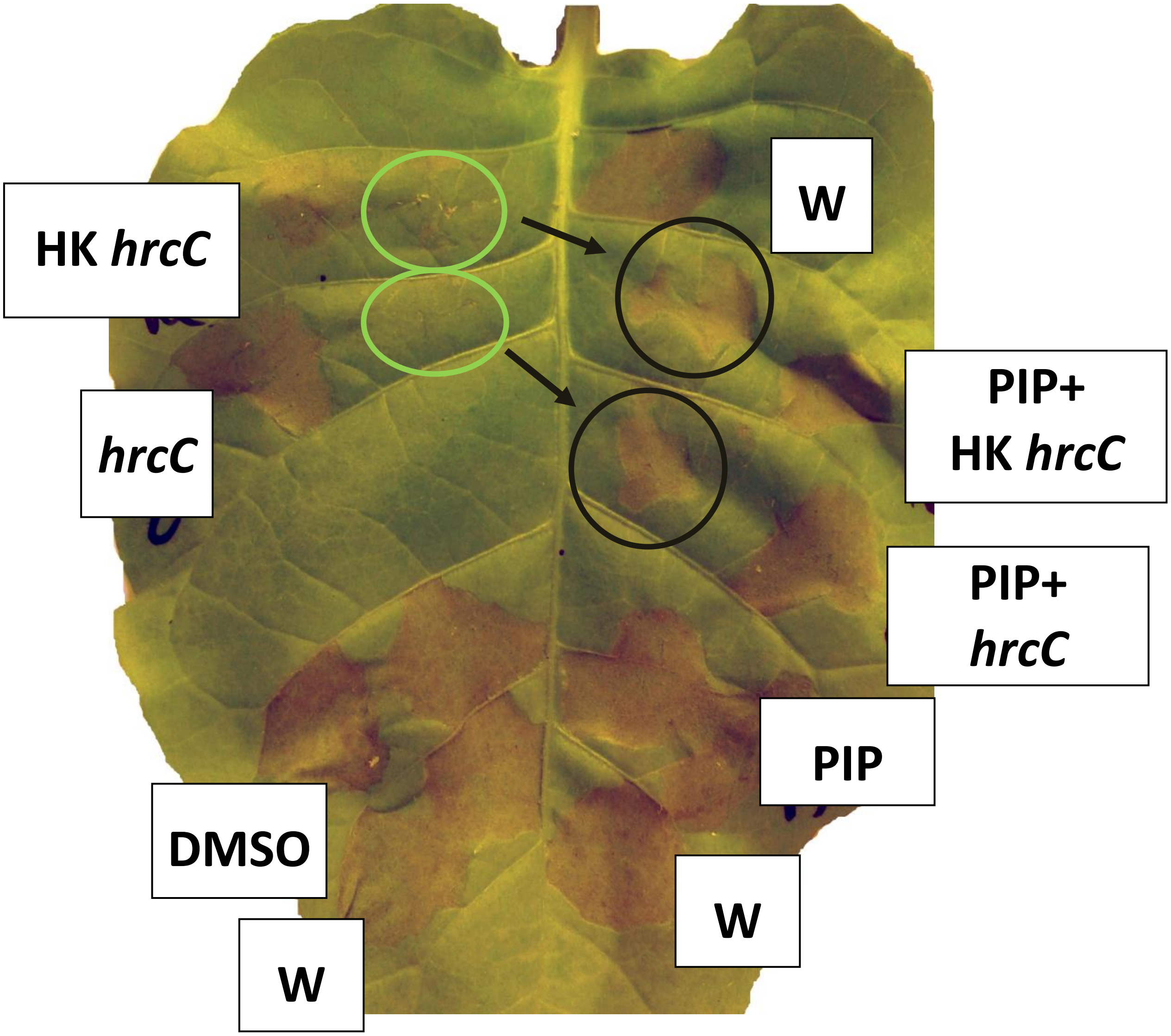

Whole intervein areas were pretreated with live *P. syringae hrcC* (hrcC) or heat killed (70°C, 15 min) *P. syringae hrcC* (HK hrcC) bacteria (5X10^{7} CFU/ml), and both were also applied in combination with piperonylic acid (PIP+hrcC and PIP+HK hrcC; with 1mM PIP diluted from 200 mM PIP in DMSO stock). Control pretreatments were piperonylic acid (PIP), dimethyl sulfoxide (DMSO), water (W). Challenge treatments with *P. syringae* 61 (HR-inducing wild type) bacteria followed after 6 hours. *P. syringae* 61 concentrations were 5X10^{7} CFU/ml along the major vein and 10^{8} CFU/ml near the leaf margin. Lighter green circling denotes weaker HR and darker brown circles denote stronger HR.
