# Supplementary material for: Pattern Triggered Immunity (PTI) in Tobacco: Isolation of Activated Genes Suggests Role of the Phenylpropanoid Pathway in Inhibition of Bacterial Pathogens
Source: PLoS One. 2014 Aug 7;9(8):e102869. doi: 10.1371/journal.pone.0102869 (PMC4125134; doi:10.1371/journal.pone.0102869)
Supplement: Information S5 — Primer sequences used for tobacco real-time RT-PCR in this work. The primers in the list were designed for Nicotiana tabacum cv. Samsun - derived gene fragments to assess expressional activation of the genes during PTI. The primers anneal optimally at 60°C and result in a PCR product of 50-150 basepairs. Therefore these primers can be run in parallel PCR reactions among the same conditions, without further optimization. (PDF) [file pone.0102869.s005.pdf]

Supporting Information S5. Primer sequences used for tobacco real-time RT-PCR in this work.
The primers in the list were designed for *Nicotiana tabacum cv. Samsun* derived gene fragments to assess expressional activation of the genes during PTI. The primers anneal optimally at 60°C and result in a PCR product of 50-150 basepairs. Therefore these primers can be run in parallel PCR reactions among the same conditions, without further optimization.

Contig/clone number	Forward primer	Reverse primer
actin	CGGAATCCACGAGACTACATAC	GGGAAGCCAAGATAGAGC
C7/EBR-15	CGT GTT GGG TTC TCT ACT CCT	GGC TGC CAA ATT GGA TGC TCT
C45/EBR-38A	TAAAACGCTTTGAACGAAGTCTA	TTAATTGGTCTCTTCTTTGCCAT
C131/EBR-38B	CAGATGATAGCAGTAGCGGAT	TGAATGTCGTCTTCTCGGGTT
C1/EBR-43	ATGATTGGAGCGACGAGCATT	GCCTCTGGAAGTATGCACTC
C5/EBR-44	TGGCTCTTGTCATCATAATCC	CCGAACCACCTCCGTTGC
C70/EBR-47	TCT TCC TTC CTC ACA TAC ATC T	ATT TTC CAA AGA CGC TTG TGG T
C154/EBR-51	GAA TGA GGA TGA ACT GAG GGA T	ATC TCA GCA GCA TTC ATA GCA T
C8/EBR-52	GGATGGAAGTAGAATCAACAAA	TTTCTTCATTCTCTGTAACTGC
C10/EBR-54	AGC AGA GCT TAG AGG TAA ACT	GCA AGA CAT CAG AGG TTA TCG T
C21/EBR-59	GATATACAACTAACGTGAAGTCAATG	TAGGAAATGCGTTCTACACTCTA
C177/EBR-61	TCG TAG CCG TTG CCG TTG 3	CGA GAG CCA ATC CGA AGG TA
C149/EBR-437	AAC GCT GAA GTA ACC CGT CAT T	GGC AAA ATC AGG AGG AGC ATA TC
C11/EBR-445	TTT CGT ATC CAA CCC GGT ATC T	GGC GGC TGC TCC AAT TAC
C3/EBR-627	ATA AAC GTA GAG GAT GCC GCT AT	CAC CTG CGT AGG AAC AAC AC
C134/EBR-635	GTC CCA GGT CAA AGG TTC TAC A	TTG CCT TCA TTT CTT CTT GTC TCT A
C24	AGCAGCTTGTTTGCTCTTCCTCAG	TGTGAGCATCCCAGGCAGTGAAAG
C53	GATTGGAGAAAGGCATCACGAC	TTTTAAGGATCCATCGCTCATCA
C108	GGA CGC TGG TGG CAT AAT C	CCT CAA GTT GAT CGC CAA GGT
C55	CCG AGC AAG CCG AGA GAT AT	GCC ACG GAG AGG AGA TTT CT
C58	TTTTCCTGACAATCCCAACATAACAGC	GCTCCACCACCATCACTTTTCTG
C59	GACCTCACTTGGACTCAATGCCAAC	GACTAGAACAAGCAGCGGAAGTGC
C66	CGA GGG CTA AAG GAA TCC A	GGT GTT GGC ACT AGC AGT CA
C90	AATTACAACAAAAACTTAACTTGCACAGGCTC	AATGTGTTCTGTATTTCAGTTGTTACCATCTC
C94	TTT GAT GAA TTG CGT TGC GT	GAT TGA ATT TTC GGG CAA ACC
C95	ATG CGT TTA ACG TGT TCG ATG	CCT GTA ATT CCT TCG CCG ATA
C106	GCT GGT GAT GTA AAG GCC GTA T	CCA AAG CCT CCA GCA TAG CA
C125	ATG CAA GGA CAA TGC GCT ATG	GCC TTC TCA TCG GTT CCC AAT
C135	AGTCAACAAAGCGCGTGAACAG	TTTCCAACATCACTTGGTGTAACAGC
C139	GGC CTT CCA ATC ACG AAC AGT	GCG TGG GCA GCA ATG ATA T
C144	TGTGGCGGAGTTAGAAAGGCATCAG	CAATCCCATTAGGCCCAGCAACAG
C151	CGGCTTTCCGACGGCGTATTG	CCAGCTTCGTTCGTCAGCTTAACAC
C39	AGCTGAATCTTGGGACTCAGTTCGTG	ACTTAAACCAGTGCTAGTGGCAACAAC
C51	CGAGGCCACAACGAGAACAATAGA	TGATCAATTCCTTCAATCGATCCA
C64	AGCAACACAACCAAGATGTGAACTC	GCTTTAAGATGTCCACGGCTTCAG
C84	GACAAATTGAGGCTGCTGCTATTATG	TGATGTTCTGAGGGCGTAACGAT
C171	CCA AGC CCA AAA AGA TCA AGC	AGC ACC ACA CTC AGC GTT AGG
